# Supplementary figures and images for: Stimulus presentation can enhance spiking irregularity across subcortical and cortical regions
Source: PLoS Comput Biol. 2022 Jul 5;18(7):e1010256. doi: 10.1371/journal.pcbi.1010256 (PMC9286274; doi:10.1371/journal.pcbi.1010256)

S1 Fig

**a**

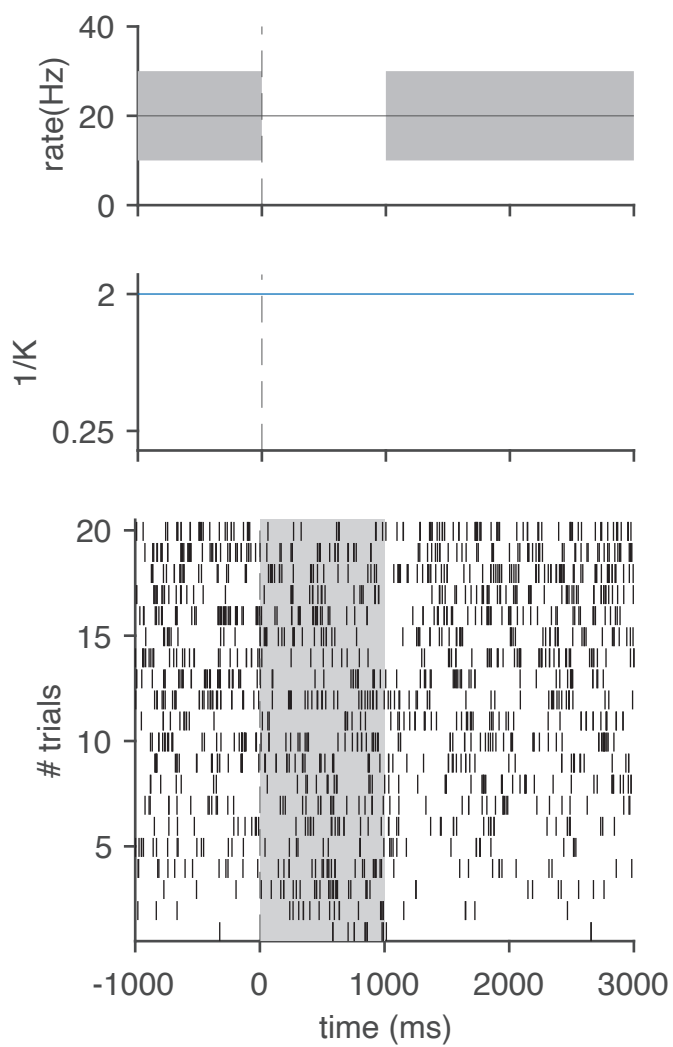

**b**

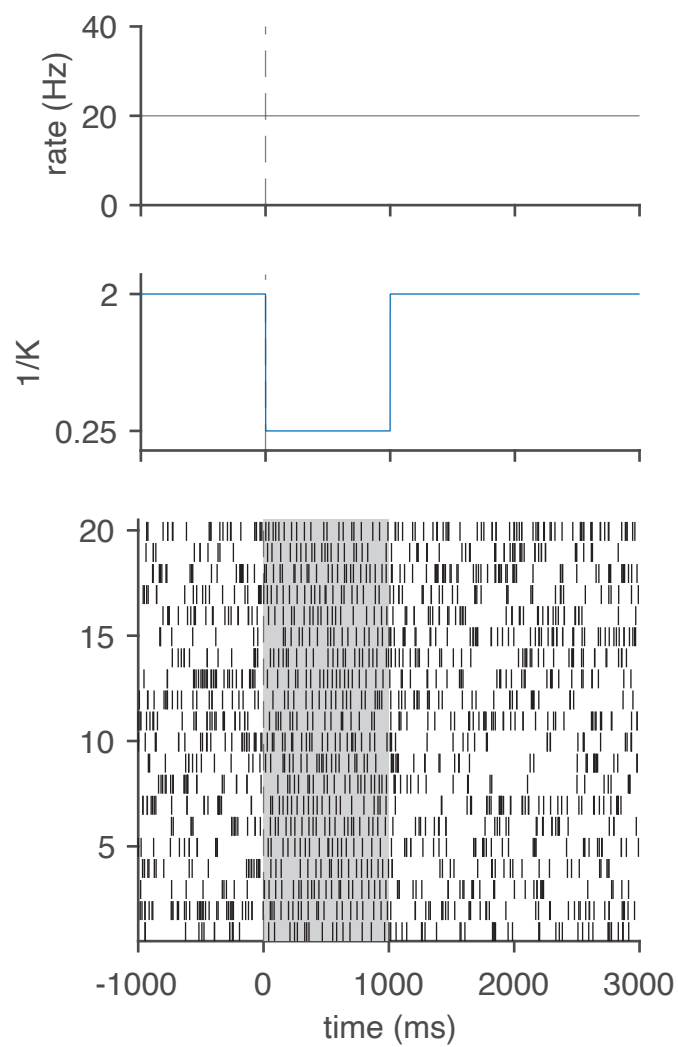

Supplement: S1 Fig — (a) A case where there was rate variability from 10–30 Hz before the stimulus and after 1 second from a stimulus onset at time zero. The rate variability collapsed to zero for 1 second after stimulus onset. Spiking irregularity was unchanged during this time (top) and the raster plot of simulated spike trains sorted by baseline rate from low to high (bottom). (b) A case where there was no rate variability during a trial but a reduction in spiking irregularity from 2 to 0.5 for 1 second period after stimulus onset (top) and the raster plot of some simulated trials. Spike generation was done with gamma inter-spiking interval where κ is the shape parameter, θ is the scale parameter and firing rateλ=1κθ. (PDF) [file pcbi.1010256.s001.pdf]

S2 Fig

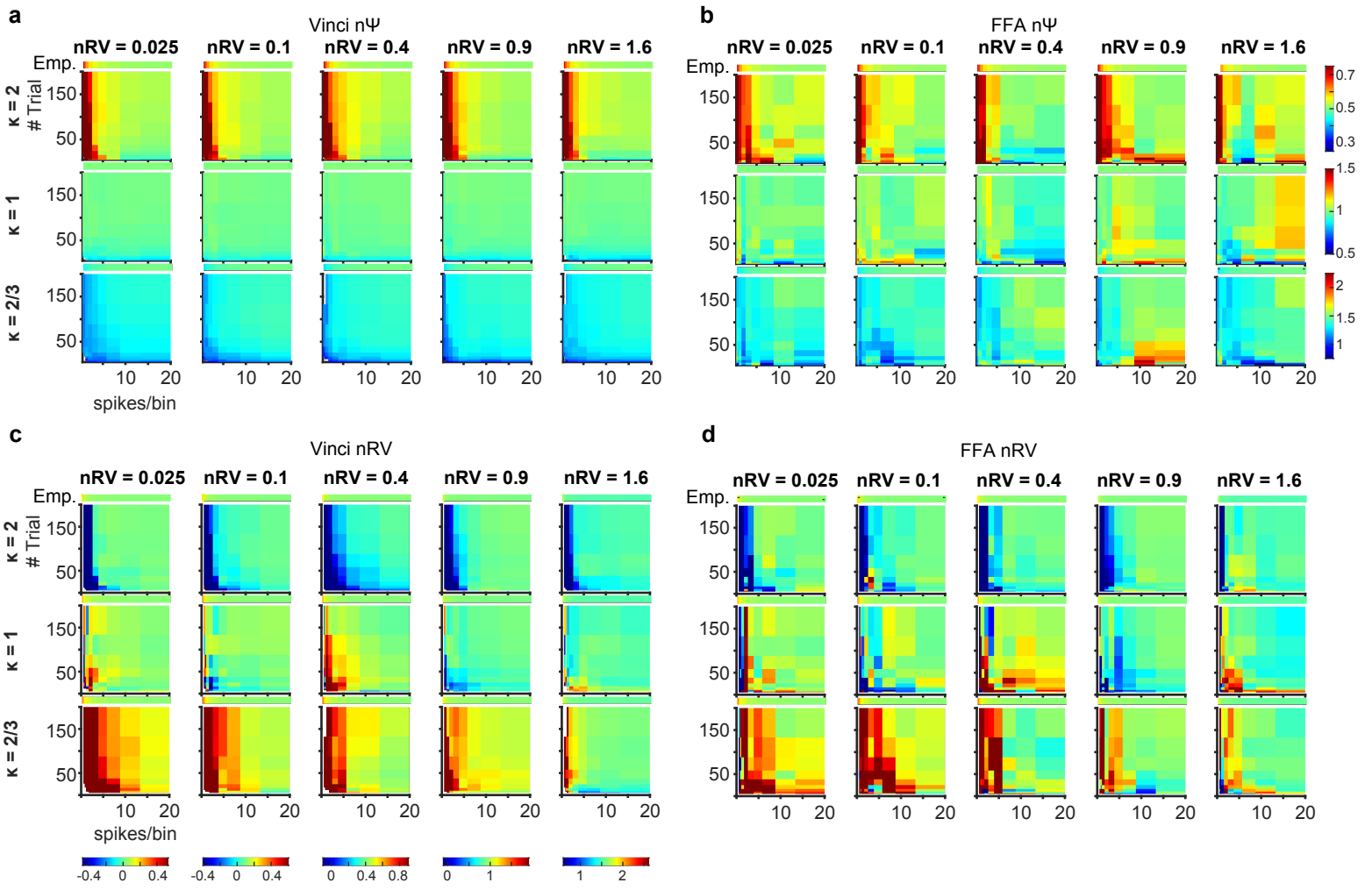

Supplement: S2 Fig — (a) Each heat map shows estimated nΨ values for a gamma processe (10Hz) for different nRV (columns) and nΨ (1κ)(rows) using the Vinci method. X-axis shows average number of spikes within time-bin and y-axis shows number of trials. Color-code is adjusted such that color green means correct estimation, while red and blue mean over- and under-estimation of the true nΨ values, respectively. The heat bar over each square heatmap indicates the empirical estimates (best possible estimate given access to many repetitions of each trial). (b-d) same format as a but for estimated nΨ values using FFA, for estimated nRV values using the Vinci method and for estimated nRV using FFA, respectively. (PDF) [file pcbi.1010256.s002.pdf]

S3 Fig

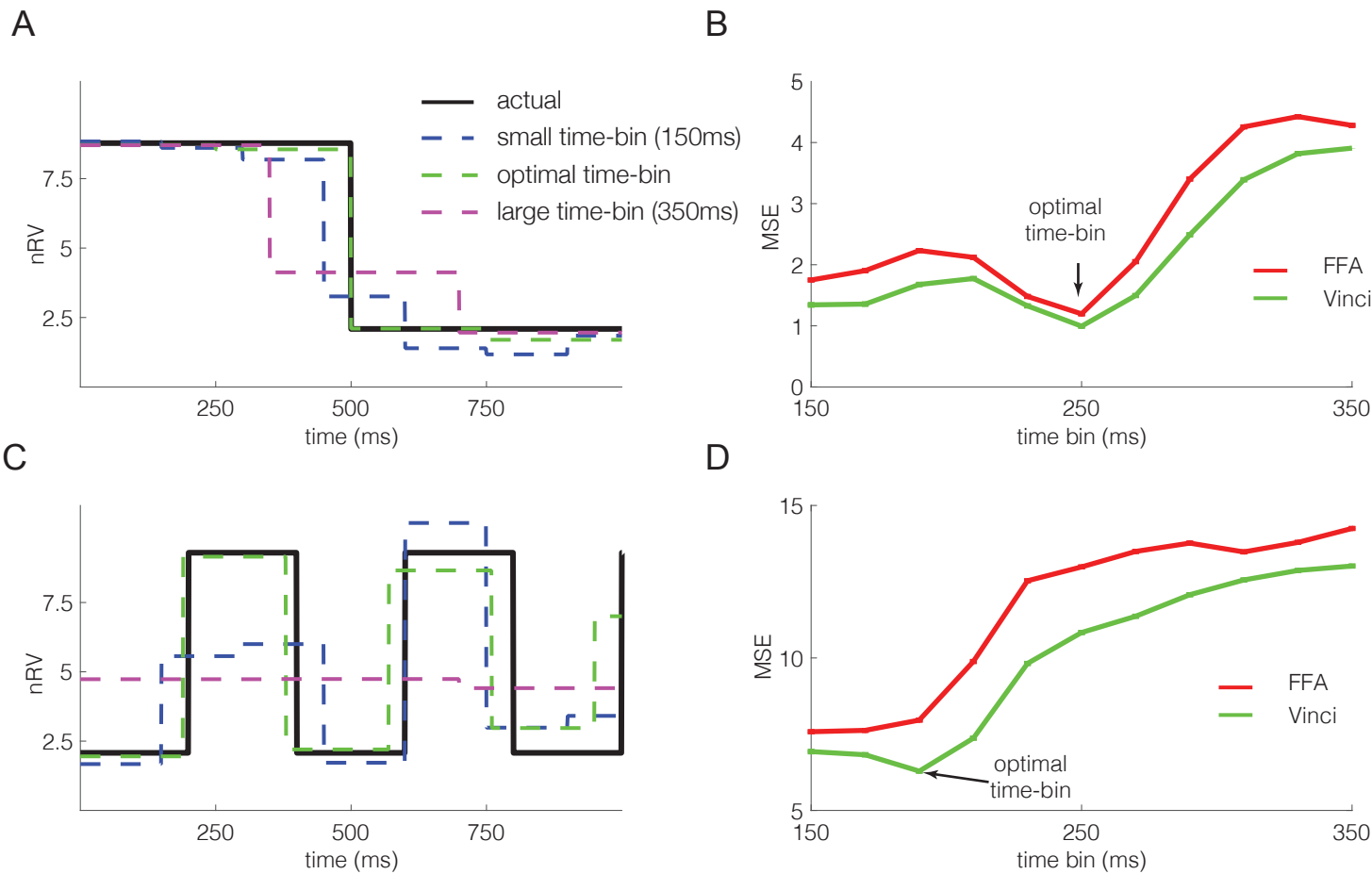

Supplement: S3 Fig — (a) Example of nRV during time with a single change from high to low during the trial (black) and the estimated nRVs using the Vinci method with different sizes of time-bins. (b) The mean sum of squared error (MSE) between the estimated nRV using FFA and Vinci methods and the true nRV by varying time-bins for the nRV pattern shown in a. The optimal time-bin (250ms) resulted in the smallest MSE compared to small (150ms) or large (350ms) time-bins. The MSE for FFA was higher than Vinci but showed a similar pattern. (c) Example of a rapidly fluctuating nRV during time from high to low during the trial (black) and the estimated nRVs using Vinci method with different time-bins. (d) The optimal time-bin (190ms) resulted in the smallest MSE compared to small (150ms) or large (350ms) time-bins for the pattern shown in c. Note the smaller optimal time-bin in this example compared to a. (PDF) [file pcbi.1010256.s003.pdf]

S4 Fig

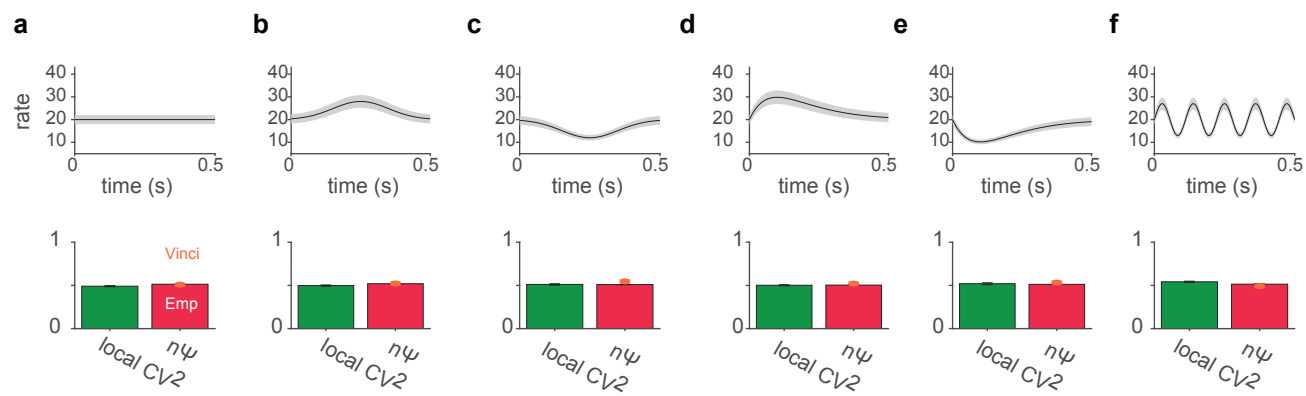

Supplement: S4 Fig — (a-f, top) firing rate pattern within a time-bin of 500ms for a gamma process with κ = 2. The shading shows firing rate variation across trials which should only effect nRV not nΨ, as in Fig 2c. (a-f, bottom) Empirical estimates of nΨ (red bar) and the CVlocal2 estimates (green bar) along with nΨ estimates by Vinci method. (PDF) [file pcbi.1010256.s004.pdf]

S5 Fig

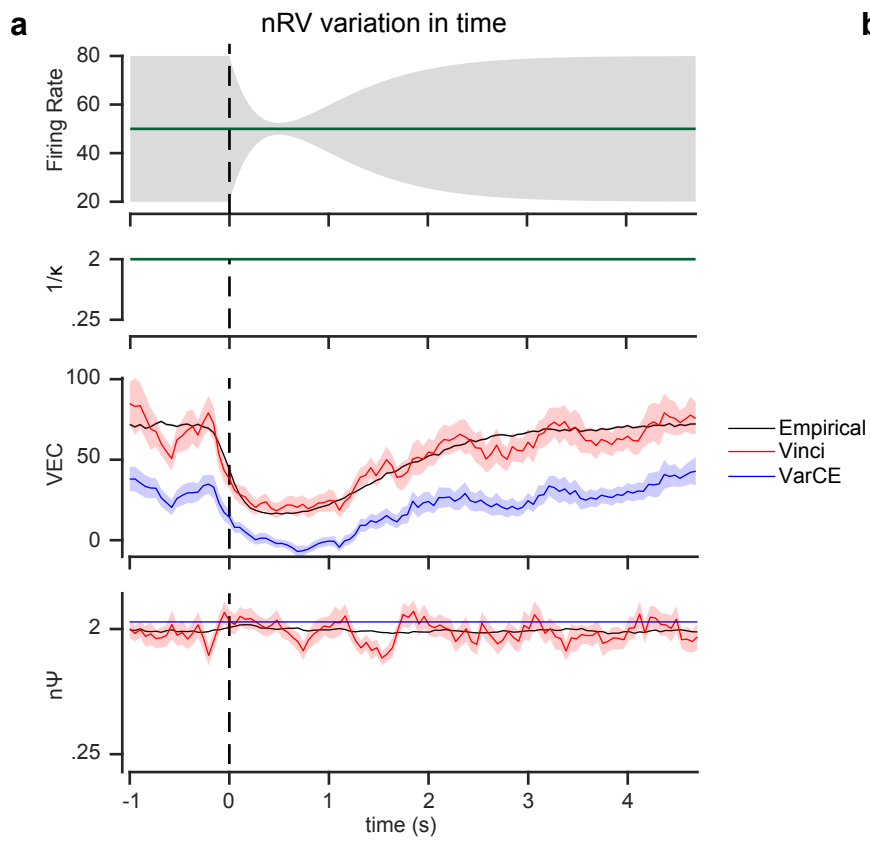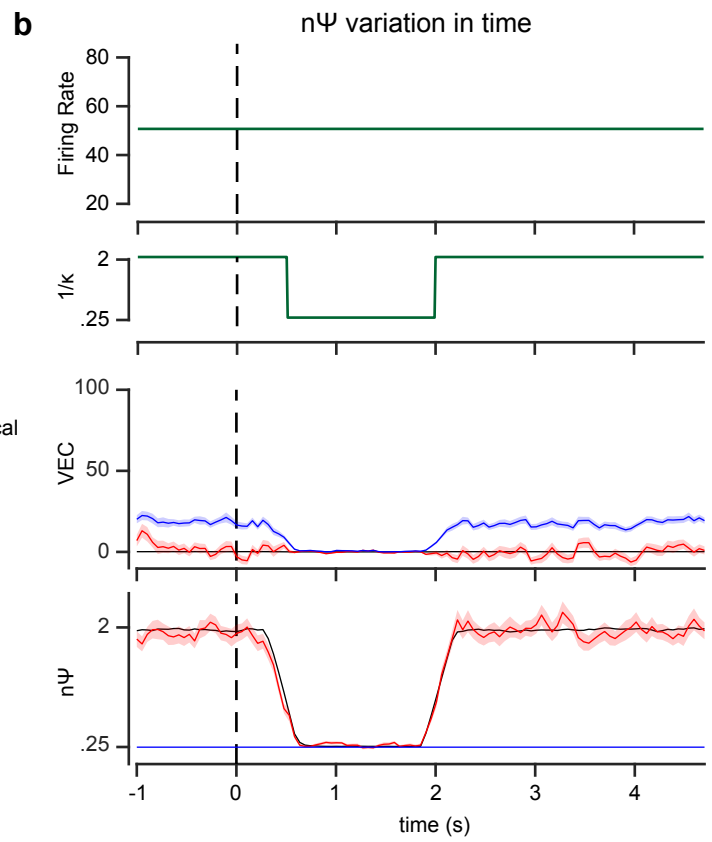

Supplement: S5 Fig — (a) Similar to Fig 1b example with a time varying nRV component but a constant nΨ component (constant κ) (top two rows). Empirical estimates of VEC and nΨ (same as ϕ) along with estimates made by Vinci and VarCE methods. (b) same format as a but for a case when there is no rate variation (VEC = T2 × RV = 0) but there is time varying spiking irregularity (changing κ). VarCE method assigns fluctuations caused by nΨ to VEC while Vinci method correctly disentangles the two different sources. (PDF) [file pcbi.1010256.s005.pdf]

S6 Fig

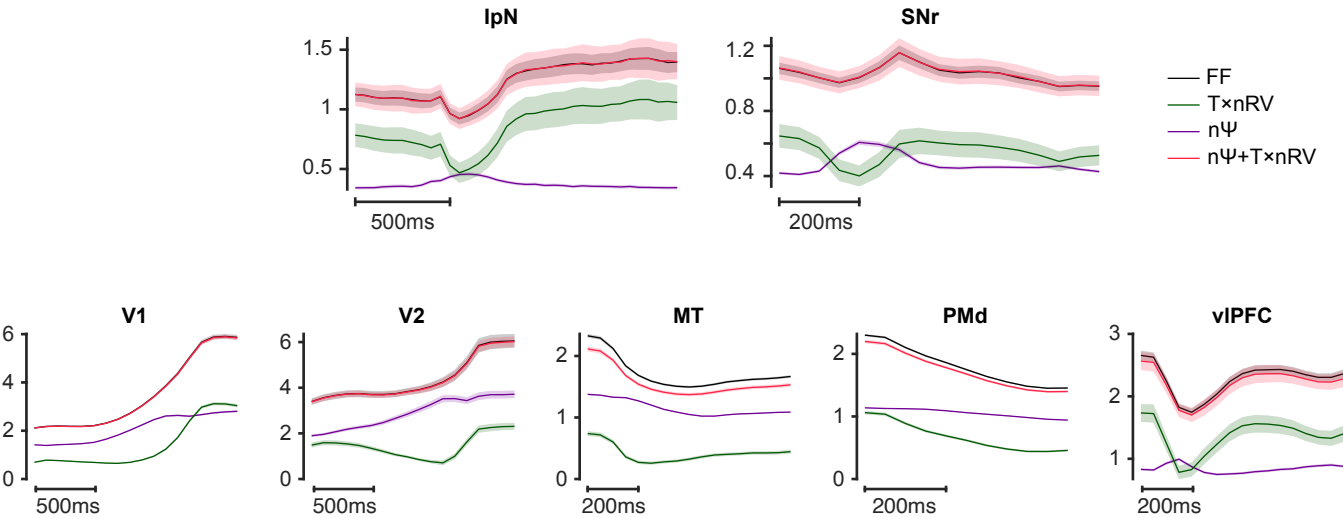

Supplement: S6 Fig — Temporal dynamics of normalized spiking irregularity nΨ and normalized rate variability nRV estimates and the fact that they sum up to almost fully explain the FF dynamics (using the FFA method). Results are shown for all subcortical and cortical regions analyzed in the main paper. The relative size and contribution of nΨ and nRV in driving the fluctuations in FF can also be examined. (PDF) [file pcbi.1010256.s006.pdf]

S7 Fig

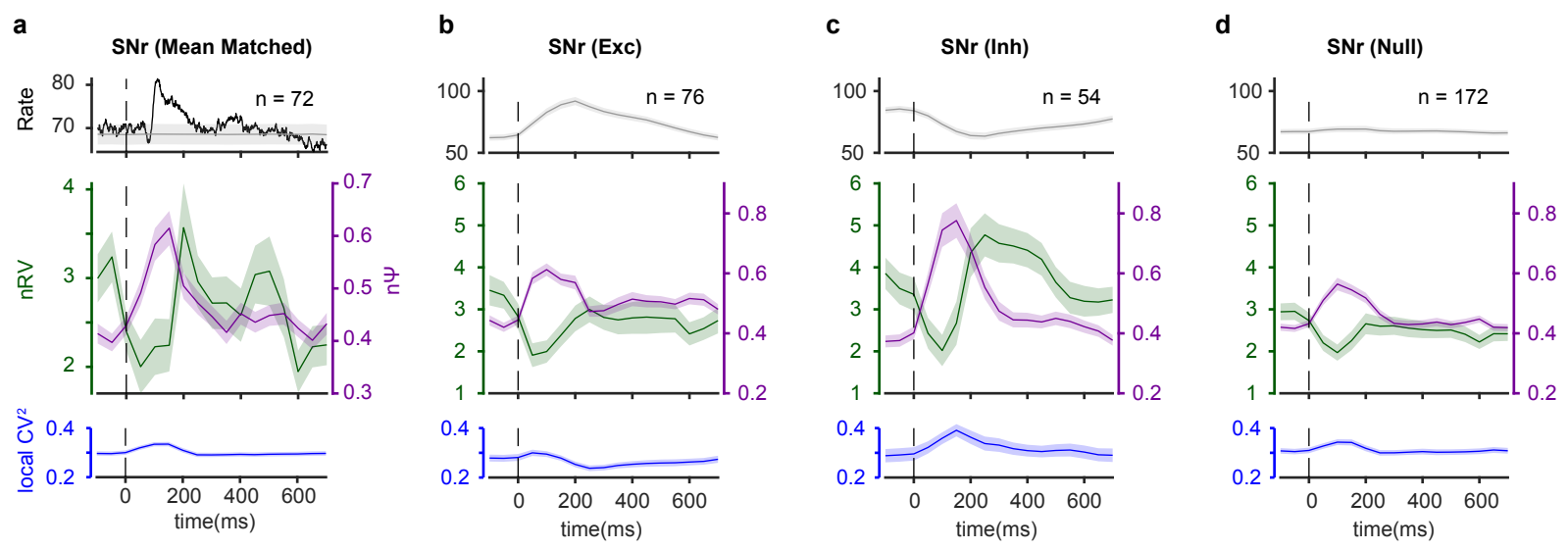

Supplement: S7 Fig — (a) Mean-matching as well as clustering the SNr neurons to three groups with (b) excitatory response, (c) inhibitory response, (d) and null response yield similar patterns in nΨ and nRV estimates as shown in Fig 4. The gray curve in (a, top) plot shows the average and sem mean matched firing using a sub-selection of neurons for each time-bin. The gray curve in (b-d, top) is the PSTH evaluated with the same time-bin (200ms) as used for variability estimates nΨ and nRV. (PDF) [file pcbi.1010256.s007.pdf]

S8 Fig

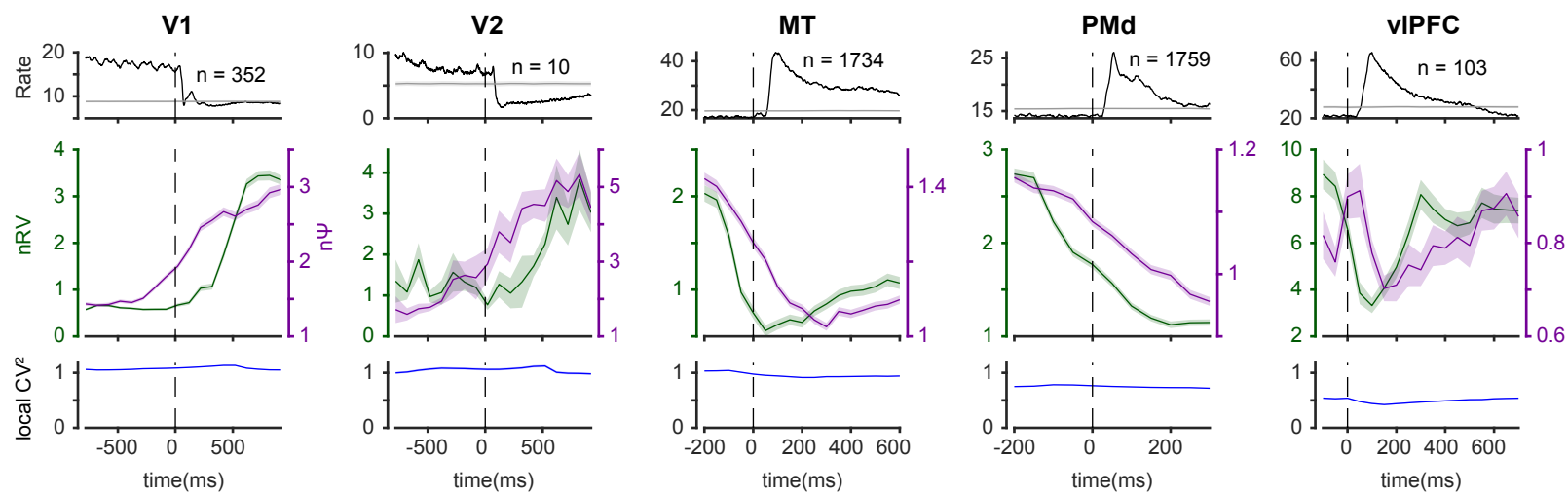

Supplement: S8 Fig — Mean matching across cortical regions yield similar patterns in nΨ and nRV estimates as shown in Fig 5. The gray curve in the PSTH plot shows the average mean-matched firing rate. (PDF) [file pcbi.1010256.s008.pdf]

S9 Fig

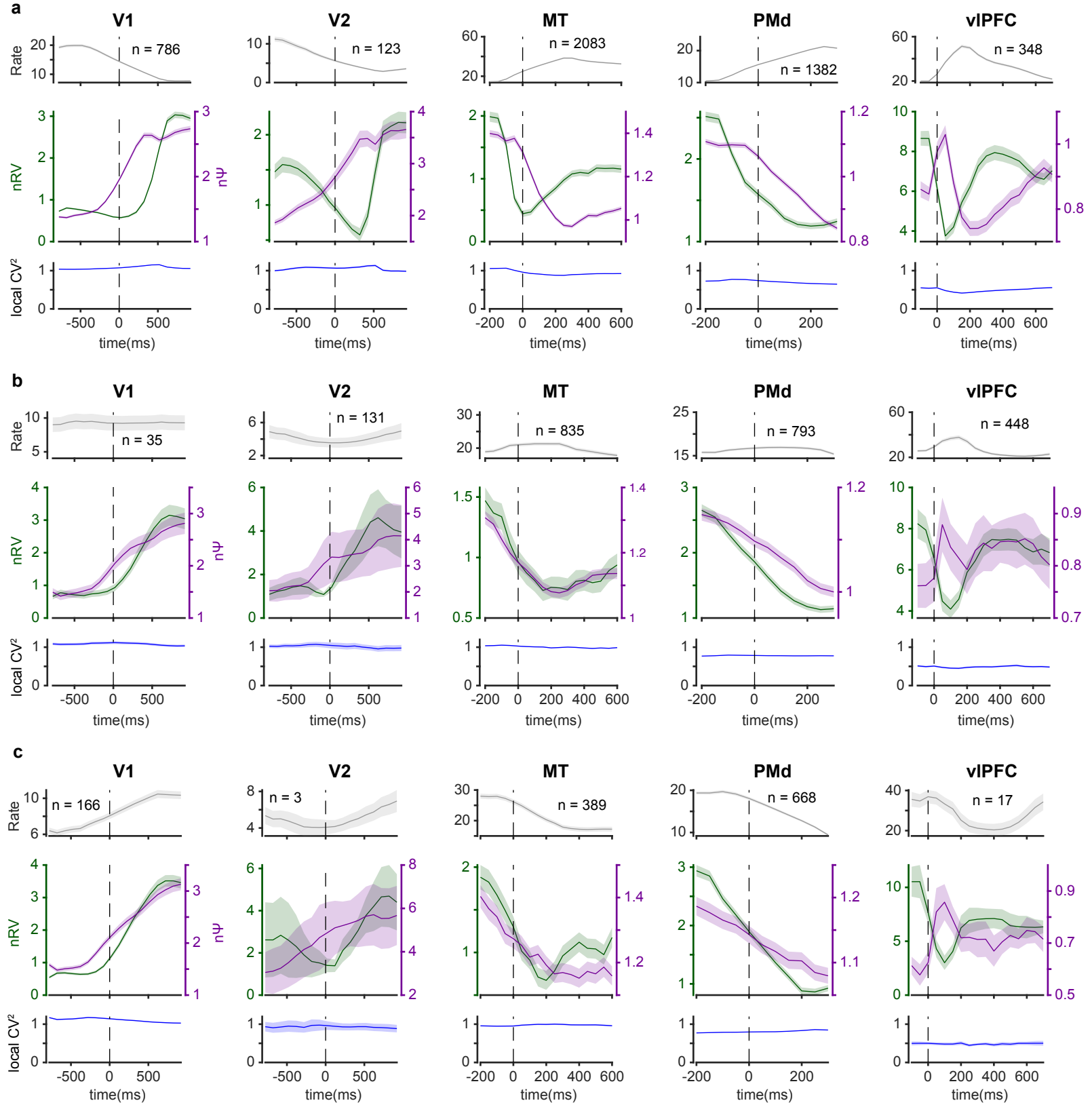

Supplement: S9 Fig — Clustering cortical neurons to three groups with (a) excitatory response, (b) null response, (c) and inhibitory response yield similar patterns in nΨ and nRV estimates as shown in Fig 5. (PDF) [file pcbi.1010256.s009.pdf]

S10 Fig

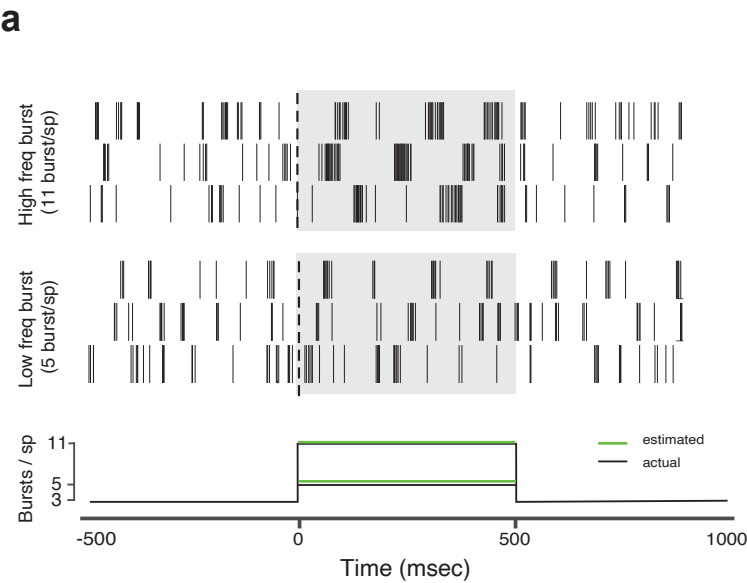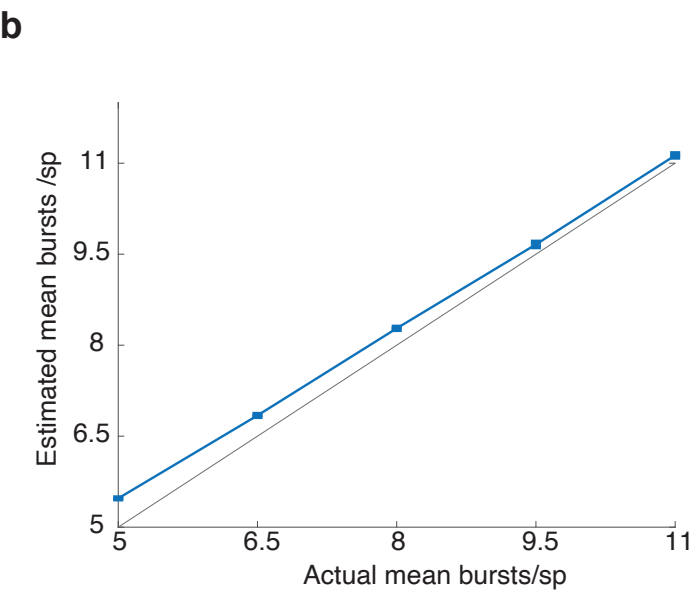

Supplement: S10 Fig — (a) Sample spike rasters with two different level of bursting activity during in 0-500ms period after the stimulus onset at zero. Burst counts from a uniform distribution with mean 11 bursts per spike (top raster) or 5 bursts per spike (bottom raster) were added with 0.5 probability to normal spikes from a gamma point process with 20Hz rate and κ = 2 shape parameter. Similar to real data, method parameters were estimated based on the spontaneous activity [–500, 0] period on a pool of 20 neurons with mean firing rate of 20Hz with a baseline burst count of 3 burst/sp (b) Mean burst count /sp estimated by the method vs the actual mean burst /sp used in generating the data. The gray line shows the unity line. (PDF) [file pcbi.1010256.s010.pdf]

S11 Fig

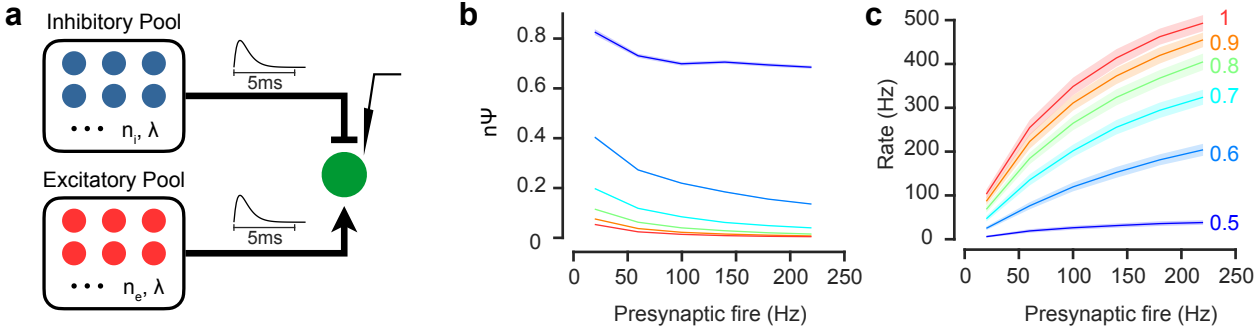

Supplement: S11 Fig — (a) Schematic of the network model including ne Excitatory (E pool) and ni Inhibitory (I pool) Poisson neurons with rate λ. The synaptic current is modeled using the same kernel for E and I connections. (b) nΨ decreases faster as a function of presynaptic rate (λ) as the color-coded excitatory ratio nene+ni approaches balanced network (i.e. 50%). (c) Postsynaptic neuron’s firing rate increases faster as a function of the presynaptic firing rate when the excitatory ratio increases. (PDF) [file pcbi.1010256.s011.pdf]

S12 Fig

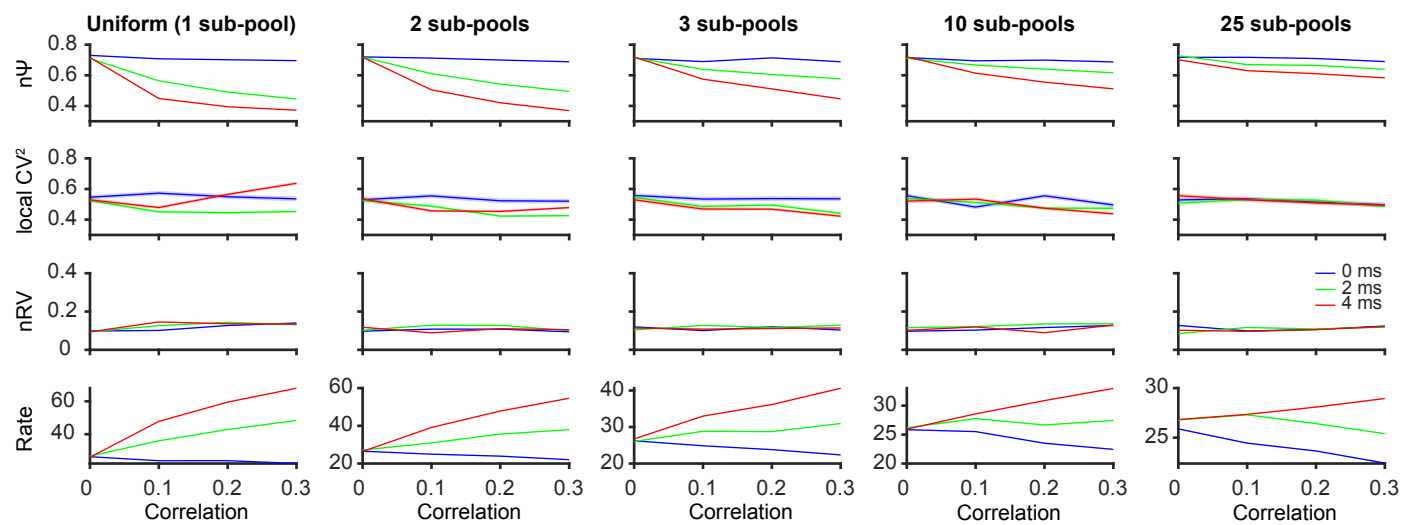

Supplement: S12 Fig — nΨ and rate sensitivity to between pool correlation decreases as we increase the number of sub-pools (for constant number of neurons). nRV and CVlocal2 remain mostly unchanged in all scenarios. (PDF) [file pcbi.1010256.s012.pdf]
