# Supplementary material for: Stimulus presentation can enhance spiking irregularity across subcortical and cortical regions
Source: PLoS Comput Biol. 2022 Jul 5;18(7):e1010256. doi: 10.1371/journal.pcbi.1010256 (PMC9286274; doi:10.1371/journal.pcbi.1010256)
Supplement: S1 Appendix — (PDF) [file pcbi.1010256.s013.pdf]

S1 Appendix : Stimulus presentation can enhance spiking irregularity across subcortical and cortical regions

Saleh Fayaz[1*], Mohammad Amin Fakharian[1,2*], Ali Ghazizadeh[1#]
[1]Electrical Engineering Department, Sharif University of Technology, Tehran Iran
[2]School of Cognitive Sciences, Institute for Research in Fundamental Sciences, Tehran, Iran
[*] equal contributions [#] Corresponding author, E-mail: ghazizadeh@sharif.edu

1 Bursting increases $n\Psi$: multiplicative and bias effects

Define: N_T : Spike count without burst, M_T : Spike count with burst. assume:

$X_T \sim binomial(N_T, P_{br})$ in which P_{br} represents probability of bursting for each spike in N_T.

Burst count (BC), for the sake of simplicity, is assumed to be constant here for each burst realization. Furthermore, we assume here there is no between trial variability or $E[N_T] = \lambda$ is same across trials.

For binomial distribution we know:

$$Var[X_T|N_T] = E[X_T^2|N_T] - E[X_T|N_T]^2 = N_T * P_{br} * (1 - P_{br}) \quad (1)$$

Furthermore:

$$E[X_T|N_T] = N_T * P_{br} \quad (2)$$

Then we have:

$$M_T = N_T + X_T * BC \quad (3)$$

$$Var[M_T] = E[M_T^2] - E[M_T]^2 \tag{4}$$

by 3 and linearity of expectation:

$$E[M_T^2] = E[N_T^2] + E[X_T^2] * BC^2 + 2 * E[N_T * X_T] * BC \tag{5}$$

By law of total expectation and 2 and 1:

$$E[X_T^2] = E[E[X_T^2|N_T]] = E[Var[X_T|N_T] + E[X_T|N_T]^2] =$$

$$E[N_T * P_{br} * (1 - P_{br}) + N_T^2 * P_{br}^2] =$$

By linearity of Expectation, finally we have:

$$E[X_T^2] = E[N_T] * P_{br} * (1 - P_{br}) + E[N_T^2] * P_{br}^2 \tag{6}$$

For the other part of the equation 5:

$$E[N_T * X_T] = E[E[N_T * X_T|N_T]] = E[N_T * E[X_T|N_T]] = E[N_T^2] * P_{br} \tag{7}$$

finally equation 5 summarize to:

$$\begin{aligned} E[M_T^2] = E[N_T^2] + (E[N_T] * P_{br} * (1 - P_{br}) + E[N_T^2] * P_{br}^2) * BC^2 \\ +2 * E[N_T^2] * P_{br} * BC = \\ E[N_T^2] * (1 + P_{br}^2 * BC^2 + 2 * P_{br} * BC) + E[N_T] * P_{br} * (1 - P_{br}) * BC^2 \end{aligned} \tag{8}$$

furthermore we have:

$$E[M_T] = E[N_T + X_T * BC] = E[N_T] * (1 + P_{br} * BC) \tag{9}$$

and

$$E[M_T]^2 = E[N_T]^2 * (1 + P_{br}^2 * BC^2 + 2 * P_{br} * BC) \tag{10}$$

Using eq 10 and 8 we can write 4 as follows:

$$\begin{aligned} Var[M_T] = (E[N_T^2] - E[N_T]^2) * (1 + P_{br}^2 * BC^2 + 2 * P_{br} * BC) \\ +E[N_T] * P_{br} * (1 - P_{br}) * BC^2 = \\ Var[N_T] * (1 + P_{br} * BC)^2 + E[N_T] * P_{br} * (1 - P_{br}) * BC^2 \end{aligned} \tag{11}$$

going back to $n\Psi_{M_T} = \frac{Var[M_T]}{E[M_T]}$ we have:

$$\begin{aligned} \frac{Var[N_T] * (1 + P_{br} * BC)^2 + E[N_T] * P_{br} * (1 - P_{br}) * BC^2}{E[N_T] * (1 + P_{br} * BC)} = \\ n\Psi_{N_T} * (1 + P_{br} * BC) + \frac{P_{br} * (1 - P_{br}) * BC^2}{1 + P_{br} * BC} = n\Psi_{M_T} > n\Psi_{N_T} \end{aligned} \tag{12}$$

2 Firing rate fluctuations have no effect on $n\Psi$:

Assume a non-stationary renewal processes with a fluctuating temporal dynamics in rate parameters $\lambda(t)$ during time-bin T. This process will have the same $n\Psi$ as any other non-stationary or stationary renewal process so long as they have equal $nPPV$ as measured by CV^2_{local} in the paper.

proof: Let's define $\Lambda(t)$ as:

$$\Lambda(t) = \int_0^t \lambda_1(t)dt$$

According to general time-rescaling theorem any point process with an integrable rate function may be rescaled into another stationary renewal point process with unit rate and equal CV^2_{local} [1,2]. Let's call the counting process for a time-bin T for the non-stationary renewal process $N(T)$ and for the corresponding rescaled stationary renewal process as $S(T)$.

For the stationary process with rate 1, for large t we have:

$$E(S(t)) = t \quad Var(S(t)) = CV^2_{local} * t \tag{13}$$

Let's define $\lambda([0,T]) \equiv \lambda(t)\,; t \in [0,T]$.For the associated non-stationary process using eq. 13 we can write:

$$\begin{aligned} E(N(T)|\lambda([0,T])) &= E(E(N(T)|\, S(\Lambda(T)), \lambda([0,T])) \\ &= E(S(\Lambda(T))) = \Lambda(T) \end{aligned} \tag{14}$$

$$\begin{aligned} Var(N(T)|\lambda([0,T])) = E(Var(N(T)|S(\Lambda(T)), \lambda([0,T])))+ \\ Var(E(N(T)|S(\Lambda(T)), \lambda([0,T]))) = \\ 0 + Var(S(\Lambda(T))) = CV^2_{local} * \Lambda(T) \end{aligned} \tag{15}$$

Similar to the derivation in [2] , we can write:

$$\begin{aligned} Var(N(T)|\lambda([0,T])) = E(Var(N(T)|S(\Lambda(T)), \lambda([0,T])))+ \\ Var(E(N(T)|S(\Lambda(T)), \lambda([0,T]))) = \\ 0 + Var(S(\Lambda(T))) = CV^2_{local} * \Lambda(T) \end{aligned} \tag{16}$$

We thus find $n\Psi_{N(T)}$ to be independant of the rate fluctuations:

$$n\Psi_{N(T)} = \frac{Var(N(T)|\lambda([0,T]))}{E(N(T)|\lambda([0,T])} = CV^2_{local} = n\Psi_{S(T)} \tag{17}$$

References

[1] Emery N Brown, Riccardo Barbieri, Valérie Ventura, Robert E Kass, and Loren M Frank. The time-rescaling theorem and its application to neural spike train data analysis. *Neural computation*, 14(2):325–346, 2002.

[2] Ira Gerhardt and Barry L Nelson. Transforming renewal processes for simulation of nonstationary arrival processes. *INFORMS Journal on Computing*, 21(4):630–640, 2009.
