# Supplementary material for: Stimulus presentation can enhance spiking irregularity across subcortical and cortical regions
Source: PLoS Comput Biol. 2022 Jul 5;18(7):e1010256. doi: 10.1371/journal.pcbi.1010256 (PMC9286274; doi:10.1371/journal.pcbi.1010256)
Supplement: S1 Text — (DOCX) [file pcbi.1010256.s014.docx]

**Supplementary Materials and Methods**

**Stimulus presentation can enhance spiking irregularity across subcortical and cortical regions**

Saleh Fayaz^1*^, Mohammad Amin Fakharian^1,2*^, Ali Ghazizadeh^1#^

^1^Electrical Engineering Department, Sharif University of Technology, Tehran, Iran ^2^School of Cognitive Sciences, Institute for Research in Fundamental Sciences, Tehran, Iran

* equal contribution

# corresponding: [alieghazizadeh@gmail.com](mailto:alieghazizadeh@gmail.com)

**Simulated spike trains:**

Spike trains in each trial were viewed as renewal point processes and ISIs were drawn from various distributions including Poisson, Gamma, and Inverse Gaussian processes (Fig 1a). Spike generation for general renewal process was done using general time rescaling theorem described in Brown et al [1]. For Fig 1b, a Gamma distribution was used. In Fig 2f bursts were added to the spike trains by adding 5 consecutive spikes with ISI = 3ms on 5% of spikes. For rate noise (Fig 2d) we generated a subsampled white noise with $\Delta t=50\mathrm{ms}$ which was added to the rate of each trial randomly. The rate switching was simulated using a Markov Process with two fast and slow rate states (Fig 2e). The transition rates from slow to fast and fast to slow state are determined by $k_{sf}$ and $k_{fs}$ respectively [2]. The process on average is in the fast state with probability $p_{f}$:

$$p_{f}=\frac{k_{sf}}{k_{sf}+k_{fs}} \left( 1 \right)$$

where we set $k_{fs}=0.005\mathrm{ms}^{-1}$ and $p_{f}=0.4$, hence the $k_{sf}\approx0.003\mathrm{ms}^{-1}$. In order to constrain the average firing rate of the total process we set the slow rate based on the desired fast and average rate. The fast rate was set to $r_{f}=30\mathrm{Hz}$ and the slow rate would be determined using the following formulation:

$$\bar{r}=p_{f}r_{f}+\left( 1-p_{f} \right)r_{s} \left( 2 \right)$$

For average rate of $\bar{r}=20\mathrm{Hz}$  the slow rate equals $r_{s}\approx13.3\mathrm{Hz}$.

Between trial rate variability (RV) was imposed by drawing rates of different trials from a gaussian distribution with different standard deviations specified for each figure (Figs 1b, 2c).

**Network simulations:**

We used LIF neuron with $N_{E}=N_{I}=50$ neurons with the following formulation [3]. We further set the parameters as in [4,5]:

$$v\left( t \right)=v_{0}-\tau\frac{dv}{dt}+rI, \left( 3 \right)$$

where $v_{th}=-55mv$, $v_{0}=-65mv$, $v_{min}=-70mv$, time constant $\tau=20ms$ with synaptic resistance of $r=80M\Omega$ and refractory period $t_{ref}=1ms$. The current $I$ was:

$$I=\left( \sum_{i}^{N_{E}} J_{i}^{E}\left( t \right)+\sum_{j}^{N_{I}} J_{j}^{I}(t) \right) \left( 4 \right)$$

where $J_{i}^{E}$and $J_{j}^{I}$ are presynaptic currents by each E/I pool neurons. The sum of currents in eq. 4 is convolved with response function defined as $te^{-\frac{t}{t_{p}}}$, $t_{p}=0.5ms$ for both excitatory and inhibitory input neurons then the resulting current will be input to eq. 3 where it would be integrated to change the voltage.

The presynaptic E/I pools (50 neurons/pool) were generated using correlated Poisson processes [6]. We added a Laplacian distributed noise to the ISIs of the generated correlated neurons with mean zero and standard deviation of 2ms. For the correlation tightness we changed the mean to 2-4ms for inhibitory neurons in order to shift inhibitory neurons after excitatory ones on average. The rates of presynaptic pools in Fig 7 at each trial are generated using the bivariate normal distribution as illustrated in Fig 7b.

**Spike count variability decomposition:**

We leveraged law of total variance and law of total expectation to decompose the spike count variability given the ISI distribution parameters in each trial $\gamma_{i}$.

$$\text{Var}\left( N_{T} \right)=\underset{\text{EVC}}{\underbrace{\text{E}\left[ \text{Var}\left( N_{T_{i}} | \gamma_{i} \right) \right]}}+\underset{\text{VEC}}{\underbrace{\text{Var}\left( \text{E}\left[ N_{T_{i}} | \gamma_{i} \right] \right)}}, (5)$$

$$\text{E}\left[ N_{T} \right]=\text{E}\left[ \text{E}\left[ N_{T} | \gamma_{i} \right] \right] (6)$$

The first term in eq.5 represent the expected variance of count (EVC) while the second term represents variance of expected count (VEC) which was termed VarCE previously [7].

We first derive eq.5 for doubly stochastic Poisson Process (exponentially distributed ISIs). For the ISIs from exponential distribution we have:

$$\text{Var}\left( N_{T} \right)=\text{E}\left[ N_{T} \right]=\lambda T (7)$$

Where $\lambda$ is the firing rate. Substituting in (5) we have:

$$\text{Var}\left( N_{T} \right)=\text{E}\left[ \lambda_{i}T \right]+\text{Var}\left( \lambda_{i}T \right)= T\times\text{E}\left[ \lambda_{i} \right]+T^{2}\times\text{Var}\left( \lambda_{i} \right) (8)$$

From (6) we have:

$$\text{E}\left[ N_{T} \right]=T\times\text{E}\left[ \lambda_{i} \right] (9)$$

Moreover, from (7-9) we have:

$$\text{FF(}N_{T})=\frac{\text{Var}\left( N_{T} \right)}{\text{E}\left[ N_{T} \right]}=1+T\times\frac{\text{Var}\left( \lambda_{i} \right)}{\text{E}\left[ \lambda_{i} \right]} (10)$$

Hence, FF for the doubly stochastic Poisson process is a linear function of time-bin $T$. As expected for stationary Poisson Process $\text{FF}=1$.

In contrast to Poisson process, the aforementioned formulations do not have a closed form for general point processes. However, for renewal processes estimates of mean spike count and its variance can be calculated in the limit of large time-bins and is shown to depend only on the mean and variance of the ISI distribution ([8], section 3.3 “The asymptotic distribution of N”). If $\text{E}\left[ \text{ISI}_{i} \right]=\frac{1}{\lambda_{i}}$ and $\text{Var}\left( \text{ISI}_{i} \right)=\sigma_{i}^{2}$, as such:

$$\lim_{T\to\infty} \text{E}[N_{T}|\lambda]=\lambda T, \lim_{T\to\infty} \text{Var}\left. \left( N_{T} | \lambda,\sigma^{2} \right. \right)=\lambda^{3}\sigma^{2}T (11)$$

By substituting (11) in (5) we have:

$$\lim_{T\to\infty}\text{Var}\left( N_{T} \right)=T\times\text{Ψ}+T^{2}\times\text{RV (12)}$$

where

$$\text{Ψ= E}\left[ \lambda_{i}^{3}\sigma_{i}^{2} \right] , \text{RV= Var}\left( \lambda_{i} \right) (13)$$

In addition, by substituting (11) in (6) the same eq.9 is derived for general renewal process. Fano factor (FF), the ratio of neural variability to its mean, is in effect a measure of neural count variability normalized by its mean. The decomposition presented in eq.12 allows one to parse FF into two components. Using eqs.9&12, one can derive the asymptotic FF for a general renewal process in relatively large time-bins:

$$\lim_{T\to\infty}\text{FF}\left( N_{T} \right)=\lim_{T\to\infty}\frac{\text{Var}\left( N_{T} \right)}{\text{E}\left[ N_{T} \right]}=n\Psi+T\times\text{nRV }$$

where

$$\text{nΨ}\text{= }\frac{\text{E}\left[ \lambda_{i}^{3}\sigma_{i}^{2} \right]}{\text{E}\left[ \lambda_{i} \right]}, \text{nRV= }\frac{\text{Var}\left( \lambda_{i} \right)}{\text{E}\left[ \lambda_{i} \right]} (14)$$

$\text{nΨ}$ and $\text{nRV}$ are normalized measures of $\Psi$ and $\mathrm{RV}$ and also intercept and slope of FF in eq.14 respectively. Note that FF and $\text{E}\left[ N_{T} \right]$ can be estimated empirically for a specified time-bin ($T$). Note that the slope term in (10) and (14) representing the nRV are identical and invariant of the distribution type. Rather, they are proportional to changes of rate parameter across trials.

The FFA method works by first estimating the FF as a function of time-bin and then by fitting its linear asymptotic behavior for which the slope represents nRV and the intercept measures n$\Psi$ (Figs 1a, c bottom, 3f, 4i, 5c). For general renewal processes n$\Psi$ is equal to $\mathrm{CV}_{\mathrm{local}}^{2}$ (proof in general case shown in S1 Appendix). For $Gamma(\kappa,\theta)$ with shape parameter $\kappa$ and scale parameter $\theta$, as in Fig 1b-e where $\theta_{i}$ but not $\kappa$ is randomly varying across trials, the equivalence of $n\Psi$ and $\mathrm{CV}^{2}$ can also be verified simply as:

$${n\Psi}_{gamma}=\lim_{T\to\infty} \frac{\text{E}\left[ \lambda_{i}^{3}\sigma_{i}^{2} \right]}{\text{E}\left[ \lambda_{i} \right]}=\frac{1}{\kappa}\frac{\text{E}\left[ \frac{1}{\theta_{i}} \right]}{\text{E}\left[ \frac{1}{\theta_{i}} \right]}=\frac{1}{\kappa}$$

$${\mathrm{CV}^{2}}_{gamma}=\frac{\text{Var}\left( \mathrm{ISI} \right)}{\left( E\left[ \mathrm{ISI} \right] \right)^{2}}=\frac{\kappa\theta_{i}^{2}}{\left( \kappa\theta_{i} \right)^{2}}=\frac{1}{\kappa} (15)$$

**Modified Vinci Method:**

The Vinci method was originally introduced for decomposition of spike count correlation (SCC) between neurons into two components firing rate correlation (FRC) and within-trial point process noise [9]. This method was shown to be able to reliably extract the latent FRC from the observed SCC by a non-parametric approach. Obviously, the problem of spike count variance of a neuron can be considered as a special case of SCC by replacing cross-correlations with autocorrelations instead. Notably, the Vinci method can be easily adapted to allow for a time-varying $n\Psi$ (aka $\phi$), unlike previous methods which either took $n\Psi$ to be equal to one (Poisson process assumption) [10] or as a tuning variable to find relative RV respectively [7]. nRV could then be calculated by subtraction of Vinci $n\Psi$ from FF following eq.14. In this paper, we refer to the $n\Psi$ and the nRV calculated in this way as the Vinci method.

Vinci method requires a set of hyperparameters: (1) K which indicates the duration in which cross-correlation between two neurons is significant (duration of significant auto-correlation in our case) (2) bin-count (m) which indicates the number of sub-bins that the time-bin is divided to (see [9]). These parameters were set manually in the original implementation. Here, for the simulated data, we chose the parameter K automatically for each neuron using its estimated shape parameter values ($\kappa$). We used eq.21 (see Local CV^2^ method) to find the shape parameter of the gamma process. Using the shape parameter and rate we first simulated a gamma process and empirically estimated the cross-correlation. K is estimated by the number of correlated lags (minimum correlation $\alpha\geq0.01$). This method can be helpful in cases where there are low number of spikes per bin or limited number of trials. We further manually tuned the K parameter for real datasets to get more stable results. In our implementation we used $m=100$.

**VarCE Method:**

VarCE finds the largest constant $\text{ϕ}$ such that VEC stays positive during the trial:

$$VEC=\text{VarCE}=\text{Var}\left( N_{T} \right)-\text{ϕ}\text{E[}N_{T}\text{]} (16)$$

We used the code provided by [7] to compute the VarCE and $\phi$.

$\mathbf{CV}_{\mathbf{local}}^{\mathbf{2}}$ **estimation:**

Several local measures of CV, as a proxy of within trial spiking irregularity, have been introduced in order to reduce sensitivity to the rate variations in time [11–15]. Theoretically for gamma process CV is only related to the shape parameter. Robust and unbiased estimation of the shape parameter could be achieved using the $\mathrm{CV}_{\mathrm{local}}^{2}$ (related to CV2, discussed below) [14,16]. Normalized point-process variability (nPPV) which represents the inherent randomness of spike generation can be quantified using inverse of the shape parameter estimate which we refer to as $\mathrm{CV}_{\mathrm{local}}^{2}$. Although the gamma distribution of ISI has previously been examined by different evaluation on fitted distribution [17,18], even in non-renewal case, like occurrence of bursting, $\mathrm{CV}_{\mathrm{local}}^{2}$ would robustly estimate the hidden gamma shape parameter [16].

We used gamma shape estimator for non-stationary gamma processes to estimate the normalized point-process variability (nPPV) based on eqs.17-18. CV2 is a measure of spiking irregularity which is robust to within trial non-stationarities [14]. $CV2\left( i \right)$ is computed by the following formulation:

$$CV2\left( i \right)=\frac{|\mathrm{ISI}_{i+1}-\mathrm{ISI}_{i}|}{(\mathrm{ISI}_{i+1}+\mathrm{ISI}_{i})/2}, (17)$$

The shape parameter of the gamma process can be estimated using CV2 as follows [16]:

$$\hat{\kappa}=\frac{2}{\left\langle\mathrm{CV}2^{2} \right\rangle}-\frac{1}{2}, (18)$$

where, $\left\langle. \right\rangle$ denotes average. We then used this unbiased estimator to calculate the nPPV of gamma process which equals $\frac{1}{\kappa}$ (eqs. 17-18) and which we refer to as $\mathrm{CV}_{\mathrm{local}}^{2}$:

$$\mathrm{CV}_{\mathrm{local}}^{2}=\frac{2\left\langle\mathrm{CV}2^{2} \right\rangle}{4-\left\langle\mathrm{CV}2^{2} \right\rangle}=\frac{1}{\hat{\kappa}} (19)$$

**Double stochasticity in the presented LIF network models:**

Approximation of network model in Figs 7-8 by simplifying assumption of integrate and fire (IF) model can be performed using Wiener process (no synaptic leakage). In this case, ISIs come from an inverse gaussian distribution with the following first and second statistical moments (for $a_{e}\lambda_{k}^{e}>a_{i}\lambda_{k}^{i}$).

$$\mu_{k}=\text{E}\left[ \text{ISI}_{k} \right]=\frac{\beta}{a_{e}\lambda_{k}^{e}-a_{i}\lambda_{k}^{i}}, (20)$$

$$\sigma_{k}^{2}\text{=Var}\left( \text{ISI}_{k} \right)=\frac{\beta\left( a_{e}^{2}\lambda_{k}^{e}+a_{i}^{2}\lambda_{k}^{i} \right)}{\left( a_{e}\lambda_{k}^{e}-a_{i}\lambda_{k}^{i} \right)^{3}}, (21)$$

where, $\beta$ represents threshold of post-synaptic IF neuron for spiking, $\lambda_{k}^{e}$ and $\lambda_{k}^{i}$ are excitatory and inhibitory firing rate of presynaptic neurons in trial $k$, and $a_{e}$ and $a_{i}$ are excitatory and inhibitory synaptic weights which are considered constant and equal to each other across trials [19].

Hence, for large enough time-bins, $n\Psi$ (or $\phi$) can be computed by substituting (23) and (24) in (14) as follows:

$${n\Psi}_{\mathrm{stoch}}=\frac{E\left[ \frac{\sigma_{k}^{2}}{\mu_{k}^{3}} \right]}{E[\frac{1}{\mu_{k}}]}=\frac{1}{\beta}\frac{E\left[ a_{e}^{2}\lambda_{k}^{e}+a_{i}^{2}\lambda_{k}^{i} \right]}{E\left[ a_{e}\lambda_{k}^{e}-a_{i}\lambda_{k}^{i} \right]}=\frac{1}{\beta}\frac{a_{e}^{2}{E[\lambda}_{k}^{e}]+a_{i}^{2}{E[\lambda}_{k}^{i}]}{a_{e}E\left[ \lambda_{k}^{e} \right]-a_{i}E\left[ \lambda_{k}^{i} \right]}, (22)$$

$${n\Psi}_{non-stoch}=\frac{E\left[ \frac{\sigma^{2}}{\mu^{3}} \right]}{E[\frac{1}{\mu}]}=\frac{1}{\beta}\frac{E\left[ a_{e}^{2}\lambda^{e}+a_{i}^{2}\lambda^{i} \right]}{E\left[ a_{e}\lambda^{e}-a_{i}\lambda^{i} \right]}=\frac{1}{\beta}\frac{a_{e}^{2}\lambda^{e}+a_{i}^{2}\lambda^{i}}{a_{e}\lambda^{e}-a_{i}\lambda^{i}}, (23)$$

$$\mathrm{CV}_{non-stoch}^{2}=\frac{\text{Var}\left( \text{ISI} \right)}{\text{E}\left[ \text{ISI} \right]^{2}}=\frac{1}{\beta}\frac{a_{e}^{2}\lambda^{e}+a_{i}^{2}\lambda^{i}}{a_{e}\lambda^{e}-a_{i}\lambda^{i}}, (24)$$

Eqs. 25-27 show that ${n\Psi}_{\mathrm{stoch}}={n\Psi}_{non-stoch}=\mathrm{CV}_{non-stoch}^{2}$ which is consistent with proof in S1 Appendix. Given simplifying assumption of $a_{e}=a_{i}=a$, we have:

$$n\Psi=\frac{a}{\beta}\frac{\nu+1}{\nu-1}, (25)$$

where, $\nu=\frac{\lambda^{e}}{\lambda^{i}}$ is the excitatory to inhibitory ratio.

Furthermore, we have:

$$\text{nRV}\text{= }\frac{\text{Var}\left( \lambda_{i} \right)}{\text{E}\left[ \lambda_{i} \right]}=\frac{\text{Var}\left( \frac{1}{\mu_{k}} \right)}{\text{E}\left[ \frac{1}{\mu_{k}} \right]}= \frac{1}{\beta}\frac{\mathrm{Var}\left[ a_{e}\lambda_{k}^{e}-a_{i}\lambda_{k}^{i} \right]}{E\left[ a_{e}\lambda_{k}^{e}-a_{i}\lambda_{k}^{i} \right]}=\frac{1}{\beta}\frac{\mathrm{Var}\left[ a_{e}\lambda_{k}^{e}-a_{i}\lambda_{k}^{i} \right]}{E\left[ a_{e}\lambda_{k}^{e}-a_{i}\lambda_{k}^{i} \right]} =\frac{{a_{e}}^{2}\mathrm{Var}\left[ \lambda_{k}^{e} \right] +{a_{i}}^{2}\mathrm{Var}\left[ \lambda_{k}^{i} \right] - 2Cov(\lambda_{k}^{e},\lambda_{k}^{i})}{a_{e}E\left[ \lambda_{k}^{e}]-a_{i}{E[\lambda}_{k}^{i} \right]} (26)$$

having other parameters of the model constant, increase in covariance (here, equivalently correlation) of $\lambda_{k}^{e}$ and $\lambda_{k}^{i}$ will decrease nRV.

**Empirical estimates of nRV and** $\boldsymbol{n\Psi}:$

In order to have a benchmark for estimators of nRV and $n\Psi$ in the simulated dataset, we used what we call the empirical estimates of these variability components. We generated a large number of realizations (~1000) of each trial with a given temporal pattern of $\gamma_{i}$ . Having access to such large number of realizations allows one to accurately estimate $\text{Var}\left( N_{T} | \gamma_{i} \right)$ and $\text{E}\left( N_{T} | \gamma_{i} \right)$ for each $\gamma_{i}$ and then calculate $\text{E}\left[ \text{Var}\left( N_{T} | \gamma_{i} \right) \right]$ and $\text{Var}\left( \text{E}\left[ N_{T} | \gamma_{i} \right] \right)$ across trials (EVC and VEC). Obviously, such empirical estimates are not available for real data but serve to set a lower bound for the error of any method which aims to use spiking data to estimate the theoretical values of nRV and $n\Psi$.

**Burst detection**$:$

For burst detection analysis, we used the Max-Interval method (MI) (Nex Technologies 2014) which was one of the recommended methods among the multiple methods discussed in Cotterill et al, 2016 [20]. This method is shown to be accurate in the presence of non-stationarities, regular bursting, noisy bursts, high frequency neural activity and in the absence of bursts. This method required 5 parameters, namely, “Maximum beginning ISI”, “Maximum end ISI”, “Minimum inter-burst interval”, “Minimum burst duration”, “Minimum spikes in a burst”. We used an automated approach to determine these parameters by first finding bursts during the spontaneous firing period across all neurons using Poisson surprise method discussed in Hanes et al, 1995 [21] which is known to work well for detecting bursts when there is no non-stationarity in the firing rates. To ensure that the parameters estimated during the spontaneous period are reasonable choices after the stimulus onset, we only used non-responsive neurons in our burst analysis shown in Fig 6 (even though MI method is supposed to handle non-stationarities).

**References:**

1. Brown EN, Barbieri R, Ventura V, Kass RE, Frank LM. The time-rescaling theorem and its application to neural spike train data analysis. Neural Comput. 2002;14: 325–346.

2. Song S, Lee JA, Kiselev I, Iyengar V, Trapani JG, Tania N. Mathematical Modeling and Analyses of Interspike-Intervals of Spontaneous Activity in Afferent Neurons of the Zebrafish Lateral Line. Sci Rep. 2018;8: 11–13. doi:10.1038/s41598-018-33064-z

3. Softky WR, Koch C. The highly irregular firing of cortical cells is inconsistent with temporal integration of random EPSPs. J Neurosci. 1993;13: 334–350. doi:10.1523/jneurosci.13-01-00334.1993

4. Van Vreeswijk C, Sompolinsky H. Chaos in neuronal networks with balanced excitatory and inhibitory activity. Science (80- ). 1996;274: 1724–1726. doi:10.1126/science.274.5293.1724

5. Gómez-Laberge C, Smolyanskaya A, Nassi JJ, Kreiman G, Born RT. Bottom-Up and Top-Down Input Augment the Variability of Cortical Neurons. Neuron. 2016;91: 540–547. doi:10.1016/j.neuron.2016.06.028

6. Macke JH, Berens P, Ecker AS, Tolias AS, Bethge M. Generating spike trains with specified correlation coefficients. Neural Comput. 2009;21: 397–423. doi:10.1162/neco.2008.02-08-713

7. Churchland AK, Kiani R, Chaudhuri R, Wang XJ, Pouget A, Shadlen MN. Variance as a Signature of Neural Computations during Decision Making. Neuron. 2011;69: 818–831. doi:10.1016/j.neuron.2010.12.037

8. Buckland WR, Cox DR. Renewal Theory. Biometrika. 1964;51: 290. doi:10.2307/2334228

9. Vinci G, Ventura V, Smith MA, Kass RE. Separating spike count correlation from firing rate correlation. Neural Comput. 2016;28: 2709–2733. doi:10.1162/NECO_a_00831

10. Goris RLT, Movshon JA, Simoncelli EP. Partitioning neuronal variability. Nat Neurosci. 2014;17: 858–865. doi:10.1038/nn.3711

11. Shinomoto S, Shima K, Tanji J. Differences in Spiking Patterns among Cortical Neurons. Neural Comput. 2003;15: 2823–2842. doi:10.1162/089976603322518759

12. Davies RM, Gerstein GL, Baker SN. Measurement of time-dependent changes in the irregularity of neural spiking. J Neurophysiol. 2006;96: 906–918. doi:10.1152/jn.01030.2005

13. Ponce-Alvarez A, Kilavik BE, Riehle A. Comparison of local measures of spike time irregularity and relating variability to firing rate in motor cortical neurons. J Comput Neurosci. 2010;29: 351–365. doi:10.1007/s10827-009-0158-2

14. Holt GR, Softky WR, Koch C, Douglas RJ. Comparison of discharge variability in vitro and in vivo in cat visual cortex neurons. J Neurophysiol. 1996;75: 1806–1814. doi:10.1152/jn.1996.75.5.1806

15. Miura K, Tsubo Y, Okada M, Fukai T. Balanced excitatory and inhibitory inputs to cortical neurons decouple firing irregularity from rate modulations. J Neurosci. 2007;27: 13802–13812. doi:10.1523/JNEUROSCI.2452-07.2007

16. Pachitariu M, Brody C, Jun P, Holmes P. Probabilistic models for spike trains of single neurons. Marius Pachitariu. Citeseer. 2015. Available: http://www.gatsby.ucl.ac.uk/~marius/papers/SpikTrainStats.pdf

17. Maimon G, Assad JA. Beyond Poisson: Increased Spike-Time Regularity across Primate Parietal Cortex. Neuron. 2009;62: 426–440. doi:10.1016/j.neuron.2009.03.021

18. Li M, Xie K, Kuang H, Liu J, Wang D, Fox GE, et al. Spike-timing pattern operates as gamma-distribution across cell types, regions and animal species and is essential for naturally-occurring cognitive states. bioRxiv. 2017; 145813. doi:10.1101/145813

19. Tuckwell HC. Introduction to Theoretical Neurobiology. Introduction to Theoretical Neurobiology. 1988. doi:10.1017/cbo9780511623271

20. Cotterill E, Charlesworth P, Thomas CW, Paulsen O, Eglen SJ. A comparison of computational methods for detecting bursts in neuronal spike trains and their application to human stem cell-derived neuronal networks. J Neurophysiol. 2016;116: 306–321.

21. Hanes DP, Thompson KG, Schall JD. Relationship of presaccadic activity in frontal eye field and supplementary eye field to saccade initiation in macaque: Poisson spike train analysis. Exp brain Res. 1995;103: 85–96.
