# Supplementary material for: Stimulus presentation can enhance spiking irregularity across subcortical and cortical regions
Source: PLoS Comput Biol. 2022 Jul 5;18(7):e1010256. doi: 10.1371/journal.pcbi.1010256 (PMC9286274; doi:10.1371/journal.pcbi.1010256)
Supplement: Attachment — Submitted filename: review_Fayaz_2021.pdf [file pcbi.1010256.s015.pdf]

Fayaz et al. introduced a new method (Fano Factor Asymptote) and improved the Vinci method for accurate estimation of two different components of neural variability: (1) across-trial rate variability (nRV) and (2) within-trial spiking irregularity (nΨ). Using these new techniques, the authors measured nRV and nΨ in neurons of cortical and subcortical regions following stimulus presentation. Contrary to previous analyses, they found that in some regions nΨ increases. Finally, they showed how similar patterns of variability can arise from balanced excitation and inhibition of a current-based LIF neuron. The article is potentially interesting but several issues should be addressed to better demonstrate the relevance and validity of the methods and analyses.

Major comments on the analyses/results:

(1) The authors demonstrate through numerical simulations how two specific network properties (rate and spike correlation between populations of neurons) affect nRV and nΨ in a post-synaptic neuron, but how can we rule out other potential mechanisms that could also give rise to similar trends in nRV and nΨ, such as all those listed at the beginning of the Discussion section? Overall, how can we reliably infer network or single cell properties from accurate estimation of nRV and nΨ given these confounding factors? If this is not possible, what are these estimates important for?

(2) The time-bin T is a key variable to understand the FFA method and should be defined and discussed in more detail, e.g. are there constraints on the duration of the time bin compared to the duration of a trial and the time scale over which the firing rate changes?

(3) When you compare results across regions, how do you control for differences in time bin? The minimum time bin that is required for the asymptotic behavior to emerge sets the maximum possible resolution for nΨ and nRV. Since the minimum required time bin is different for different regions, the inferred nΨ and nRV must have different resolution. What is the precise relationship between time bin and resolution, and between time bin and properties of the neural activity, such as the firing rate? Is the firing rate the only factor that determines the time bin of asymptotic behavior?

(4) In the case of Fig.1, there are no other sources of nΨ other than nPPV. However, the rate changes within each trial in Fig.1. Although the rate doesn't undergo abrupt state switching, I would expect slightly smoother state switching to give a non-nPPV component of nΨ similarly to the case shown in Fig.3f. It would be helpful if the authors could provide some intuitions for why steep versus smooth state switching give qualitatively different results.

(5) Why do nRV and nΨ follow the same stimulus-induced changes in neurons that do not respond to the stimulus (Supplementary Fig.S5)?

(6) Do the results in Fig.7 depend on the relative size of the subpools? For example, how do the results change when increasing the size of the subpools from 0 to the size of each entire pool of excitatory/inhibitory neurons? How do the results depend on the relative size of the excitatory versus inhibitory pools/subpools?

Clarity issues:

(1) Pag.8, the authors wrote: “Using the FFA method, one would be able to track the temporal fluctuations in nΨ and nRV during a trial”: this is a bit confusing, because nRV is defined as the variability across many trials; I think this concept could be better explained as tracking fluctuations in nΨ and nRV over time (within and across trials, respectively).

(2) Fig1d caption: “nRV estimates made empirically by assuming access to a large number of realizations given constant parameter”: if parameters are constant, nRV is identically zero. To my understanding, realizations with constant parameters are useful to estimate nΨ, not nRV. Please clarify.

(3) Please specify how you computed correlation between firing rates in Fig.6 and how it differs from the correlation in Fig.7. In Fig.6, are you considering correlations (between the excitatory and inhibitory pools) in firing rates averaged over each trial?

(4) I didn't get what Fig.6c represents: in what way is the presynaptic firing rate non-random?
(5) Labels pool #1/pool#2 in Fig.7b don't correspond to neither pool1/pool2 nor subpool1/subpool2 in Fig.7a. Please clarify.
(6) What is the spike delay in Fig.7f? How do the results for within-pool spike correlation depend on spike delay?
(7) What does "[0,1] ratios of time-bin" mean in Fig.1 caption?
(8) The authors discuss some results on conductance-based neurons as being consistent with observations in V1, V2, MT, PMd. Where are these results shown?

Questions on the Methods/Supplementary:
(1) I'm confused by the signs in eq.(3). The correct LIF eq. is $\tau \frac{dv}{dt} = -v(t) + v_0 + rI$
(2) It's not clear how the synaptic current response function is defined (below eq.4): isn't the synaptic current integrated over time?
(3) Would you please clarify how you got eq.(11), right?
(4) In the Modified Vinci Method, you mentioned “in our implementation we used m=100”: what is m?

Typos:
There are several typos/grammar errors, e.g. “evidence suggest”, “our results cautions”, “the changes in spiking regularity still has”, “gamma processe”, “green mean correct estimation”, ...
