# Supplementary material for: Stimulus presentation can enhance spiking irregularity across subcortical and cortical regions
Source: PLoS Comput Biol. 2022 Jul 5;18(7):e1010256. doi: 10.1371/journal.pcbi.1010256 (PMC9286274; doi:10.1371/journal.pcbi.1010256)
Supplement: Attachment — Submitted filename: Response to Reviewers_submission.pdf [file pcbi.1010256.s016.pdf]

Dear Editor,

We would like to thank you for coordinating the review process and the reviewers for their important and insightful comments. We have addressed reviewers' comments below and have indicated the resulting modifications to the manuscripts. Major changes in the manuscript text are highlighted. As the result of comments, we have now added 1 main and 3 supplemental figures as well as one Appendix containing proofs to the manuscript.

Regards,
Saleh Fayyaz, MohammadAmin Fakharian and Ali Ghazizadeh
21-Feb-2022

Reviewer1:

Comments to Author

> This paper discusses the problem of variability partitioning in neural spike trains. It presents a method for decomposing variability in trial spike irregularity and trial-by-trial rate variability that can also be used in cases where the trial spike irregularity changes over time. The method is compared with an existing approach, and together they are applied to experimental and simulated data. The chief conclusion from the analysis of experimental data is that external stimuli can increase spike irregularity in some brain areas, and can in certain cases counteract the reduction in variability that is usually expected to follow stimulus presentation.
>
> Overall, the conclusions from the experimental analyses presented in this paper are interesting, and in my opinion the simulations do a good job of providing minimal examples of mechanisms that could give rise to certain empirical trends. However, I found the way in which the FFA and Vinci methods are presented rather confusing or misleading (see below). I cannot recommend the paper for publication in its present form, but I may reconsider my recommendation if the paper was revised to address my concerns.

We are grateful to the reviewer for the important comments. We have partly rearranged our narrative as a result of the comments as indicated below.

[Major points]

1. My main concern with this paper is that the proposed method (FFA)

seems strictly inferior to the Vinci method, except for pedagogical purposes. If it is introduced just for the sake of explanation, then this should be made clear from the beginning, so that the reader doesn't go through the introduction and the initial part of the results thinking that this is a novel contribution that solves the problem of variability partitioning better than existing approaches. Otherwise, if FFA is supposed to stand on its own, the authors have to provide a clear motivation as to why a reader would want to use it, in practice, rather than the Vinci method. At present, such motivation seems missing from the paper.

We thank the reviewer for the important comment. Our main goal here was to show that the presumption of a constant within trial spiking variability can result in erroneous conclusions when dealing with real data. This goal entailed at least two steps: first was the introduction of an intuitive way to present the various sources of variability and visualize their distinct effects (not a strong point for Vinci method). This was done by using the FFA which clearly shows the variability effects on its slope and intercept However, as we explicitly mentioned trying to estimate nΨ and nRV by directly fitting FFA may not be the best numerical approach. For that purpose, we used an adaptation of the method proposed in Vinci et al 2016 to find a robust measure of EVC from which nΨ was then calculated. FFA results are mainly presented in later figures to validate the overall agreement between Vinci nΨ and the intercept in FFA as well as the resulting nRV.

Note that in both methods the decomposition derived for fano factor in eq.2 is used to derive nRV and thus fano factor calculation and derivations presented in the beginning of result section is important in both approaches. The original Vinci method and our modifications to it is described in detail in Supplementary Method section under the heading 'Modified Vinci Method'

To address the reviewers comment we have now reorganized our introduction and results section in multiple places to clarify that we only recommend using the Vinci method for variability analysis. Some of these changes are now reflected in Introduction section page 3:

"Here, we present a principled approach that allowed us to find the absolute value of VEC and track changes in ϕ and to further breakdown its constituents. Briefly our approach works by explicitly relating FF to normalized RV and Ψ (nRV and nΨ). nΨ which is the same as ϕ is estimated by invoking minimal assumptions for a doubly stochastic point process by adapting an existing method that allowed relatively accurate estimation of nΨ (*19*). Given FF and nΨ the remainder of variability will be due to nRV."

and Result section pages 8-9:

"In practice finding the nΨ and nRV by fitting the asymptotic behavior of FF as a function of time-bin while intuitive is not the best practice if quantitative minimum variance estimators are desired (e.g. nΨ estimates can be noisy as small change in slope translate to large swings in the intercept).

Therefore, we used a more robust estimator of EVC using an existing nonparametric approach developed by Vinci et al (referred to as Vinci method, see Supplementary Methods) (*19*). Once the EVC is estimated one can normalize it by $\langle N_T \rangle$ which is the average of N_T across trials (as one's best estimate of $\mathrm{E}[N_T]$) to estimate nΨ. Given FF and nΨ one can then estimate nRV using eq.2. This method allows for a more robust estimation of nΨ and nRV (Figure 1d-e). Note that both FFA and Vinci methods estimation require sufficiently large number of trials and spikes per time-bin to give accurate estimates of nΨ and nRV. However as noted, the Vinci estimate is more robust compared to FFA across a range of parameters and should be the method choice when analyzing neural data (Figure S2)."

In absence of a solid answer to this question, the reader would be better served by a paper simply about the modified Vinci method and its application to experimental and simulated data (and therefore with more details on that technique, which at the moment are rather sparse - even after reading the supplement, the reader is left with almost no insight as to how the Vinci approach works, and how does it look in its "modified" form), with a separate appendix or discussion section devoted to the explanation of the FFA perspective, corredated by a schematic like Figure 1 and some comparisons like Figure S1 that show that this method is inferior to Vinci. Alternatively, FFA could be removed entirely from the paper, and published elsewhere in the context of a tutorial paper on variability partitioning.

We have already had a full section in the method describing the Vinci method and the way it works (Modified Vinci Method in Supplementary Methods section). We have now added more details on the method in that section as following:

"The Vinci method was originally introduced for decomposition of spike count correlation (SCC) between neurons into two components firing rate correlation (FRC) and within-trial point process noise (*19*). This method was shown to be able to reliably extract the latent FRC from the observed SCC by a non-parametric approach. Obviously, the problem of spike count variance of a neuron can be considered as a special case of SCC by replacing cross-crorrelations with autocorrelations instead. Notably, the Vinci method can be easily adapted to allow for a time-varying nΨ (aka ϕ), unlike previous methods which either took nΨ to be equal to one (Poisson process assumption) (*1*) or as a tuning variable to find relative RV respectively (*4*). We then calculated nRV from the subtraction of Vinci nΨ from FF following equation 14. In this paper, we refer to the nΨ and the nRV calculated in this way as the Vinci method."

Some of the other modifications we have done are added and highlighted in that same section.

In addition, we have added some simple explanation of the way Vinci method is used in the result section page 8-9 to present the reader with some basic understandings without the need to read the detailed methods (the same paragraph pasted to the previous question, which we quote the relevant part from here again)

"Therefore, we used a more robust estimator of EVC using an existing nonparametric approach developed by Vinci et al (referred to as Vinci method, see Supplementary Methods) (*19*). Once the EVC is estimated one can normalize it by $\langle N_T \rangle$ which is the average of N_T across trials (as one's best estimate of $E[N_T]$) to estimate nΨ. Given FF and nΨ one can then estimate nRV using eq.2. This method allows for a more robust estimation of nΨ and nRV (Figure 1d-e)."

2. I was unable to access the data and code repository at, as the DOI does not resolve.
Apologies for the broken link. The data and code are now available at:
https://www.ghazizadehlab.org/index.php/resources/

[Minor points]

1. I believe the paper would be enhanced by the addition of a schematic with some simple raster plots (either cartoon or from a simulation) as a figure in the introduction, showing what nΨ and nRV differences look like visually. This would greatly enhance the readability of the paper for readers that may not be familiar with the general concept of variability partitioning (or that may just need a reminder). I'm thinking of something like the raster plots in Figure 7, which do a great job of showing the possible components of the correlation. This is just a suggestion however.

Thank you for the suggestion. Indeed, our Fig 1 was created to serve this exact purpose (i.e. to visually show the components of variability in raster plots). To address the comment, we have added a new supplementary Fig S1 which shows the effect of nΨ and nRV separately on simulated spike trains.

2. When introducing the Vinci method, please use a more explicit phrasing; "a previously introduced method" could be construed as implying that this method is due to the same authors, when it's not; something like "an existing method" is less ambiguous and would work better.

We have changed the reference to the Vinci method in the introduction as "by adapting an existing method that allowed relatively accurate estimation of nΨ (*19*)"

Reviewer: 2
Comments to Author
Fayaz et al. introduced a new method (Fano Factor Asymptote) and improved the Vinci method for accurate estimation of two different components of neural variability: (1) across-trial rate variability (nRV) and (2) within-trial spiking irregularity (nΨ). Using these new techniques, the authors measured nRV and nΨ in neurons of cortical and subcortical regions following stimulus presentation. Contrary to previous analyses, they found that in some regions nΨ increases. Finally, they showed how similar patterns of variability can arise from balanced excitation and inhibition of a current-based LIF neuron. The article is potentially interesting but several issues should be addressed to better demonstrate the relevance and validity of the methods and analyses.

We are grateful to the reviewer for the important comments which we have addressed below.

Major comments on the analyses/results:

1- The authors demonstrate through numerical simulations how two specific network properties (rate and spike correlation between populations of neurons) affect nRV and nΨ in a post-synaptic neuron, but how can we rule out other potential mechanisms that could also give rise to similar trends in nRV and nΨ, such as all those listed at the beginning of the Discussion section? Overall, how can we reliably infer network or single cell properties from accurate estimation of nRV and nΨ given these confounding factors? If this is not possible, what are these estimates important for?

The reviewer raises an important point. Indeed, there could be a large number of possible mechanisms that give rise to certain temporal patterns in nRV and nΨ and our simulations are not to be construed as the exclusive mechanisms to explain the observed dynamics in neurol data. However, we note that our main goal in this paper was not to enumerate the possible mechanisms that control neural variability but to provide a better understanding and an accurate methodology for estimation and parsing components of variability which does not rely on erroneous assumptions on constant spiking irregularity as done by most previous publications in the field and by doing so we have uncovered stimulus induced changes in variability that was missed by previous methods such as VarCE.

Obviously, while a more accurate estimation of neural response variability does not by itself identifies a unique underlying neural or network mechanisms, it can result in a meaningful reduction of the possible mechanisms by ruling out processes that *cannot* produce the observed dynamics in variability components.

These points are not added to the discussion section page 30 lines 2-7:

"We also note that our main goal in this paper was not to enumerate the possible mechanisms that control neural variability but to provide a better understanding and an accurate methodology for estimation and parsing components of variability and by doing so we have uncovered stimulus induced changes in variability that was missed by previous methods such as VarCE that assume a constant nΨ within a trial. Obviously, while a more accurate estimation of neural response variability does not by itself identifies a unique underlying neural mechanisms, it constrains them to those that give rise to the temporal dynamics observed in each of nΨ and nRV."

2- The time-bin T is a key variable to understand the FFA method and should be defined and discussed in more detail, e.g. are there constraints on the duration of the time bin compared to the duration of a trial and the time scale over which the firing rate changes?

The time-bin T is indeed an important parameter in both Vinci and FFA methods (or for any method for that matter as shown by dependance of Empirical estimates in Fig S2). We believe current Fig S2 addresses the comment raised by the reviewer. Here we show the performance and dependance of both FFA and Vinci methods on the number of trials used for estimation as well as the number of spikes per time-bin T. As expected in both cases the increase in number of trials and number of spikes per bin resulted in better estimation both nΨ and nRV. This figure was described in multiple places in the result section and discussed in a full paragraph in the discussion section page 28 lines 13-22:

"Figure S2 shows that the need for having high a temporal resolution in estimating nRV and nΨ should be balanced by the requirement to choose a large time-bin with enough spikes (>5 spikes/bin) for estimation accuracy and thus may not be readily useable for regions with very low firing rates if high temporal resolution is required. Indeed, for regions with low firing rate estimates of nRV and nΨ maybe under and over-estimated respectively for small time-bins. Most of the areas examined in this study had sufficiently high firing rates but we may be prone to some misestimation for area V2 which had low firing <5Hz during stimulus off time (Figure 5). Note that the requirement for the sufficient number of spikes per time-bin is due to the nature of doubly stochastic point process and exists even for idealized simulations with access to a large number of trial repetitions with the same parameter (i.e. empirical estimate, Figure S2). The problem of low spike counts can be partially addressed by using model-based approaches and additional assumption on smoothness of firing rate changes during a trial but with some loss of generality (*71*)."

3- When you compare results across regions, how do you control for differences in time bin? The minimum time bin that is required for the asymptotic behavior to emerge sets the maximum possible resolution for nΨ and nRV. Since the minimum required time bin is different for different regions, the inferred nΨ and nRV must have different resolution.

As discussed in the previous point, the temporal resolution of our estimates is inversely related to the size of time bin. On the other hand we need to use a larger time bin to have enough spikes

for accurate estimations (as discussed in point 2). Our choice for analysis shown in Figs 3-5 was to choose the smallest possible bin size that still gave accurate results for each region based on the simulations in Fig S2.

This means that the resolution of our estimates for different regions were different (generally higher for high firing subcortical areas such as SNr and lower in low firing cortical neurons). If the strict comparison across regions are required, one then is bounded to use the lowest resolution across all regions.

The exact size of the time-bin used for each region is mentioned in the Fig 3-5 captions but are mentioned here for ease of checking:
IpN: 300ms , SNr: 200ms, V1: 1000ms, V2: 1000ms, MT: 400ms, PMd: 400ms, vlPFC: 200ms

What is the precise relationship between time bin and resolution, and between time bin and properties of the neural activity, such as the firing rate? Is the firing rate the only factor that determines the time bin of asymptotic behavior?

As mentioned above the larger the time bin the lower the resolution. Also, higher firing rates allows choosing smaller time bins as discussed in point 2 and Fig S2. Apart from the paragraph mentioned in the discussion, we have an explanation of this in Result section page 6 line 20-21:

"Also note that while in theory FF asymptotic behavior is at infinity limits of time-bin, in practice the linear asymptotic behavior may be observable with much smaller time-bins depending on the firing rate of the neuron (Figure 1a, S2)."

4- In the case of Fig.1, there are no other sources of nΨ other than nPPV. However, the rate changes within each trial in Fig.1. Although the rate doesn't undergo abrupt state switching, I would expect slightly smoother state switching to give a non-nPPV component of nΨ similarly to the case shown in Fig.3f. It would be helpful if the authors could provide some intuitions for why steep versus smooth state switching give qualitatively different results.

This was an astute observation by the reviewer of a subtle but interesting point. We were originally of the idea that white noise or rapid state switching may inflate nΨ beyond nPPV. This was despite some preliminary proofs that we had in the previous version of the paper (eq 16 and eq25-27 in the previous version) which indicated that non-stationarities should not cause such an inflation under some approximations.

However, as we were checking our spike generation function for non-stationary renewal processes for this comment, we found a bug which caused error in spiking intensities specially at low rates and rapid changes (briefly the error was with faulty implementation of time-rescaling theorem). We have now fixed that bug and rechecked our spike simulation codes against a similar code that our colleague Hideaki Shimazaki generously sent us to ensure accuracy. As a result, we can now verify that noise and state switching are not causing nΨ to inflate beyond nPPV and have

modified our Fig 2 (previously Fig 3) accordingly. We have modified the result section page 10 accordingly:

"On the other hand, bursting (*30*) which is a clear violation of renewal assumption (i.e. history dependance to previous spike occurrences beyond ISI parameters) can be theoretically shown to inflate nΨ beyond nPPV (Appendix I) and accordingly results in larger spiking irregularity beyond nPPV (Figure 2f). Notably, such inflation of empirical nΨ is not observed even when there is significant non-stationarities such as rate noise (*34*) or rate switching (*7*, *35*) (Fig 2d-e) consistent with theoretical predictions (Appendix I) (*36*). Note however that while Vinci and FFA tolerate random rate noise, they seem to slightly overestimate nΨ in rate switching with random and rapid changes from low to high firing across trials."

To further confirm this finding, we have now done a formal mathematical proof of this phenomena which we are presenting in the newly added Appendix I. There, we present the result that the nΨ of any renewal process will only depend on its nPPV regardless of its rate being stationary or not.

This proof clearly limits the sources of nΨ inflation to factors that cause nonrenewality such as seen for bursting (Fig 2f). We have now added burst detection for our neural data and can verify that bursting pattern in at least some regions match the changes of nΨ beyond nPPV. We have now added Fig 6 and supplemental Fig S9 to address the bursting issue.

5- Why do nRV and nΨ follow the same stimulus-induced changes in neurons that do not respond to the stimulus (Supplementary Fig.S5)?

The phenomenon of changing variability in nonresponsive neurons is reported previously (Churchland et al 2010, Nature , Fig 5). In Churchland et al the authors provide an intuition for this using a simple neural network (their Supplementary Fig 1). The idea is that the presence of the stimulus stabilizes the network by putting all neurons (responsive and nonresponsive) in a less variable state across trials. Interestingly, we observe that while nRV is reduced as predicted by the aforementioned explanation, nΨ increases after the onset in all neuron types in the SNr. Indeed, in our simulations in figure 7 we have exactly observed this phenomenon. In this case changes in the rate correlation in inh and exc input neurons resulted in reduction of nRV and a mild increase in nΨ (Fig 7d) but had no effect in the firing rate of the output neuron. As mentioned before this increase in nΨ can be manifested by increased burstiness of the neurons (Fig 6)

6- Do the results in Fig.7 depend on the relative size of the subpools? For example, how do the results change when increasing the size of the subpools from 0 to the size of each entire pool of excitatory/inhibitory neurons? How do the results depend on the relative size of the excitatory versus inhibitory pools/subpools?

We already had the answer to the reviewer's question in Fig S11 (previously Fig S8). There we have looked at the sensitivity of the result as we increased the number of subpools (which effectively reduces the overall correlations within excitatory and inhibitory pool, as there are more independent units in each pool). As expected, the larger the number of subpools, the smaller the reduction on nΨ. If by relative size of pools the reviewer means examining the network in the out of balance condition (where excitatory neurons exert higher influence than inhibitory neurons) we have now added Fig S10 examining that effect in the case where there is no correlation among pools.

Clarity-issues

1- Pag.8, the authors wrote: "Using the FFA method, one would be able to track the temporal fluctuations in nΨ and nRV during a trial": this is a bit confusing, because nRV is defined as the variability across many trials; I think this concept could be better explained as tracking fluctuations in nΨ and nRV over time (within and across trials, respectively).

   We have now clarified the sentence in page 8 as following "Indeed by examining the FF asymptote (FFA), one would be able to track the temporal fluctuations in nΨ and nRV over time using a sliding time window"

2- Fig1d caption: "nRV estimates made empirically by assuming access to a large number of realizations given constant parameter": if parameters are constant, nRV is identically zero. To my understanding, realizations with constant parameters are useful to estimate nΨ, not nRV. Please clarify.

   For the empirical estimates using simulated data, we assume that one has access to a large number of realizations of a given trial to allow for correct estimation of ISI parameters in that trial and then a large number of trials to measure the between trial variability of rate (VEC and nRV) and the expected value of count variance (EVC and nΨ). Calculating this empirical measure is important because it shows the lower bound of error for any method which claims to estimate VEC and EVC as the true values of parameters are never available for real data.

   We have now added a full section for empirical estimate to supplementary method section under the heading:

   "**Empirical estimates of nRV and nΨ:**

   In order to have a benchmark for estimators of nRV and nΨ in the simulated dataset, we used what we called the empirical estimates of these variability components. We generated a large number of realizations (~1000) of each trial with a given temporal pattern of θ_i . Having access to such large number of realizations allows one to accurately estimate $\mathrm{Var}(N_T|\theta_i)$ and $\mathrm{E}(N_T|\theta_i)$ for each θ_i and then calculate $\mathrm{E}[\mathrm{Var}(N_T|\theta_i)]$ and $\mathrm{Var}(\mathrm{E}[N_T|\theta_i])$ across trials (EVC and VEC). Obviously, such empirical estimates are not

available for real data but serve to set a lower bound for the error of any method which aims to use spiking data to estimate the theoretical values of nRV and nΨ."

3- Please specify how you computed correlation between firing rates in Fig.6 and how it differs from the correlation in Fig.7. In Fig.6, are you considering correlations (between the excitatory and inhibitory pools) in firing rates averaged over each trial?

Please note figure numbers change from 6-7 to 7-8.
In figure 7 correlation refers to the between trial correlation in parameters in this case the firing rate or λ_i. While in figure 8 we created spike correlation using the method introduced in "C. Gómez-Laberge, A. Smolyanskaya, J. J. Nassi, G. Kreiman, R. T. Born, Bottom-Up and Top-Down Input Augment the Variability of Cortical Neurons. *Neuron* **91**, 540–547 (2016)." Briefly, here we create a template spike train and then with some probability keep the spike to create spiking correlation while keeping the average rate constant. Then introduce a shift to the trains to model the delay.

To clarify the differences, we have now added a description of this in page 23 of Result section:

"The presynaptic E/I pools (50 neurons/pool) were generated using correlated Poisson processes (Macke et al., 2009). We added a Laplace distributed noise to the ISIs of the generated correlated neurons with mean zero and standard deviation of 2ms. For the correlation delay we changed the mean to 2-4ms for inhibitory neurons in order to shift inhibitory neurons after excitatory ones on average. Note that in this case the correlation exists both within each E/I subpool and between corresponding E/I subpools. "

4- I didn't get what Fig.6c represents: in what way is the presynaptic firing rate non-random?
Figure 7c (previously 6c) shows that *postsynaptic* firing rate does not change as the function of between pool rate correlation across trials. Indeed, it remains to the condition in which the presynaptic rates are deterministic (as shown in Fig 7c).

5- Labels pool #1/pool#2 in Fig.7b don't correspond to neither pool1/pool2 nor subpool1/subpool2 in Fig.7a. Please clarify.

Thanks for pointing this out. We have now fixed this. In Fig 8a we call both groups in exc and inh subpool#1 and then write subpool#1 in Fig 8b. Other correlated subpools like subpool#2 is also denoted in Fig 8b.

6- What is the spike delay in Fig.7f? How do the results for within-pool spike correlation depend on spike delay?

In Fig 8d-f, spike delay is not defined because we are considering correlation to only happen within exc and inh pools and not between them. (The spiking delay in Fig 8a-c is between pools)

What does "[0,1] ratios of time-bin" mean in Fig.1 caption?
This was a typo and removed (in older version we showed a normalized time bin but now Fig 1a show the actual duration in the x-axis)

7- The authors discuss some results on conductance-based neurons as being consistent with observations in V1, V2, MT, PMd. Where are these results shown?
We referred to paper by S. Cavallari, S. Panzeri, A. Mazzoni, Comparison of the dynamics of neural interactions between current-based and conductance-based integrate-and-fire recurrent networks. *Front. Neural Circuits* **8**, 1–23 (2014) in a few sentences below. This paper showed differences in CV sensitivity in current based vs conducatnce based models based on preynaptic rates. But since we have not observed the presynaptic rates in our neurons and the argument was not central to our findings, we decided to remove this part in the new version of the paper and only cite that paper as a possibility to explain some of the differences observed across

Questions on the Methods/Supplementary:

(1) I'm confused by the signs in eq.(3). The correct LIF eq. is $\tau dt = -v(t) + v\,0 + rI$
Thank you. We corrected the equation in new manuscript.

(2) It's not clear how the synaptic current response function is defined (below eq.4): isn't the synaptic current integrated over time?
"The sum of currents in eq. 4 is convolved with response function defined as $te^{-\frac{t}{t_p}}$, $t_p = 0.5ms$ for both excitatory and inhibitory input neurons then the resulting current will be input to eq. 3 where it would be integrated to change the voltage." This explanation is now added to the method section.

(3) Would you please clarify how you got eq.(11), right?
This part is taken from ref (30) (section 3.3 "The asymptotic distribution of N"). We have added this additional info in the method section.

(4) In the Modified Vinci Method, you mentioned "in our implementation we used m=100": what is m?

As we mentioned in the manuscript: "bin-count (m) which indicates the number of sub-bins that the time-bin is divided to (see (19)".

In Vinci method, first we divide each time-bin to 'm' sub-bins. nΨ, for each time-bin, is then calculated using estimations on those sub-bins. The detailed explanation of main

method can be find in the “G. Vinci, V. Ventura, M. A. Smith, R. E. Kass, Separating spike count correlation from firing rate correlation. *Neural Comput.* **28**, 2709–2733 (2016).”

We have a detailed explanation of the Vinci method and our modifications in method section under the heading ‘Modified Vinci method’.

Reviewer #3: Fayaz et al present a high-quality paper that provides a possible solution to more accurately quantify the multiple sources of variability in single neurons’ spiking. The key issue is that variability (e.g. measured by for example Fano factors) arises due to several factors: across trial variability and ~spike irregularity. Previous results have lumped these sources of variability together, though they are different, and the information about network states that one can infer from them is distinct. I think this paper is a very good fit for PLOS COMP BIOLOGY and represents a systematic advance.

We are grateful to the reviewer for the positive view of this work and for the comments which we have addressed below.

I have several conceptual suggestions which are important to consider when thinking about noise across many brain areas that should make it into the Discussion.

For a long time now, changes in variability in subcortical brain areas have been observed (often showing different changes than in "sensory cortex" cortex, (see studies of medial septum, e.g., old studies by Buzsaki showing neural rhythms for example in the septohippocampal system that occur across large groups of neurons relative to state changes). There are many reasons for the differences between variability in sensory cortex versus other areas, that I think are worth acknowledging and carefully discussing in the framing of the method.

Brain areas (cortical and subcortical) have different intrinsic timescales, may integrate information differentially relative to external stimuli (sensory events) and internal stimuli (e.g. state changes). And there broadly put: we don’t know what a “stimulus” is for many brain areas (versus V1 or A1 which have clear stimuli that can be used to systematically drive neurons and study across trial, within trial, and across neuron variability). Because we do not know what is a "stimulus" for many brain areas we do not know how to correctly interpret across trial variability - which may not be variability at all but a tight tracking of an internal state (internal stimulus) that we do not understand. Again, while we know how to drive a V1 neuron we may not really understand the high dimensional

"RF" (if that is even a right concept for PFC) of a "vlPFC" neuron. So, with that in mind I think some deep conceptual limitations in across area comparisons in this and other studies of variability need to be acknowledged.

The reviewer raises deep and important points. Indeed, from discussion with a few of our colleagues who have experience recording in subcortical areas, most of acknowledged the observation about the irregular spiking pattern in areas such as basal ganglia, cerebellum and septum and the notable differences with cortical areas (and in particular sensory cortexes) reported in the literature. In this context the point the reviewer raised is very interesting. It maybe that the temporal fluctuations in the animal internal state as a function of stimulus onset can affect non sensory areas such as the limbic circuitry to violate renewal assumptions and create within spiking irregularities in ways that are not observed normally in stereotypical cortical computations needed for sensory processing.

We have now added a paragraph discussing this point and the limitations of cross area comparisons with regard to the issue of variability in pages 27-28:

"It is important to note that cross area comparison of spiking variability should be done with care as the meaning, the origin and the physiological function of spiking variability across different regions could be different. While spiking irregularity can be considered noise in the rate code scheme it may be the signal if a region is using temporal code. Temporal code is observed in many subcortical areas (*65*) including hippocampus (*66*) ,septum (*67*) cerebellum(*68*) superior olivary nucleus(*69*) while at least in some cortical areas rate code may be dominant(*43*, *70*). In addition, the role of variability in sensorimotor corteces vs associative corteces and subcortical areas may be different as we do not know or cannot always control what is considered the 'true' stimuli for such regions. In other words, spiking variability observed as nRV and/or nΨ in associative corteces and subcortical areas may well be sensitive to complex interactions between high level features of stimuli, internal states, attention and motivation that are hard to observe and control but are nevertheless different from encoding noise. "

Minor

Please define vlPFC precisely. As the last author knows, vlPFC is many brain regions (eg., 46, 46, 12, etc). Please be precise in what is vlPFC.

The vlPFC data is taken from Ghazizadeh et al 2018 current biology paper and includes 8Av, 46v, and 45. We have now added this info in the method section.
