# Supplementary material for: Stimulus presentation can enhance spiking irregularity across subcortical and cortical regions
Source: PLoS Comput Biol. 2022 Jul 5;18(7):e1010256. doi: 10.1371/journal.pcbi.1010256 (PMC9286274; doi:10.1371/journal.pcbi.1010256)
Supplement: Attachment — Submitted filename: Reviewer_comment_R2.pdf [file pcbi.1010256.s017.pdf]

Dear Colleagues,

Once again, we would like to thank the editor and the reviewers for their constructive comments on this manuscript. As a result of the new comments, we have now added a paragraph in the discussion and a new supplementary figure. A few citations are added to the paper as the result of changes. Below, we have addressed the two remaining points of reviewer #2.

Ali Ghazizadeh & Saleh Fayyaz

Reviewer #2: The authors addressed some important issues, but I still have two concerns.

(1) I understand that the goal of the paper is not to enumerate the possible mechanisms that control neural variability, but to provide a more accurate method for parsing components of variability. However, the authors confirm that the purpose of parsing components is to rule out processes that cannot produce the observed dynamics in variability. Given the very large number of possible mechanisms that give rise to the same variability components, from the molecular to the cellular to the network level, can we even rule out some of the possible mechanisms? The example using a LIF neuron model provided by the authors does not answer the question of what mechanisms are ruled out. If the goal of accurately parsing variability is to infer properties of the neurons or neuronal networks, it would be important to show a concrete example in which this is possible. Alternatively, could you provide a clear explanation (including specific examples) for why is it important to refine estimates of variability components, and what can we learn from these accurate estimates?

We thank the reviewer for raising this point which we think merits some clarification. As we have mentioned in the first line of introduction, consistent changes in spiking variability in response to events can be an additional mechanism by which information about the environment are encoded in the neuronal activity beyond the changes in mean firing rate. Therefore, any benefit that one can enumerate for accurate estimation of instantaneous firing rates (say by using PSTHs) in encoding and decoding information also applies to accurate estimation of instantaneous firing rate variability. That is accurate estimation of spiking statistics affected by external stimuli and behavioral events is in and of itself a valuable end in neuroscience as it reveals the information encoding/decoding in neurons and is the "main purpose" of this paper.

Constraining the underlying neural mechanisms is a secondary benefit of accurate estimation of changes in firing rate statistics (be it first order statistics in firing rate PSTH or second order statistics like variability). Observing excitatory response in a neuron bears evidence for certain types of inputs (e.g. Glutamatergic) and against others (e.g. GABAergic). Along the same lines, we have shown that given an LIF model (which we know is a poor approximation of reality), one can use the behavior of nSI and nRV components to favor one scenario over others. For instance, observing increased nSI and constant nRV might indicate changes in within pool correlation while observing decreased nSI and constant nRV favors changes in between pool correlation (Fig 8). Of course, the same way that mean firing PSTH is insufficient for pinpointing the underlying neural

circuitry so is the firing variability. But both mean firing and its variability carry crucial information that is indispensable for any attempt to constrain neural mechanisms.

To address the reviewer's comment and to further clarify the main goal of this paper we have now added the following paragraph to the discussion page 26-27:

"Accurate estimation of different components of spiking variability, allows one to reveal neural strategies for encoding information beyond mean firing rate and on the decoding side opens new possibilities to decipher information hidden in neural activity about external stimuli and internal processes by an ideal observer. For instance, observing between trial variability across tasks and time periods has been discussed as another source of neural information coding, besides the averaged neural response (*1*, *3*, *4*, *6*, *11*, *17*). Also, different patterns of neural variability in cortical and subcortical regions have been found recently (*17*) which may point to different encoding strategies in these regions. Subcomponents of neural variability, also, has been shown to convey information about the stimulus and neurons. For instance, nPPV has been shown to separate neurons in terms of working memory coding efficiency in prefrontal cortex (*32*). Parietal neurons seem to have the same nPPV across various tasks (*66*)."

(2) As regards point 2 in the response letter, I appreciate the clarification on how the number of spikes per time bin affects the method accuracy, but my question was how does the relative duration of the time bin compared to the duration of the trial (or the number of time bins per trial) affect accuracy? One could increase the time-bin duration to increase the number of spikes per time bin, but this would give rise to a trade-off in accuracy (as the authors also acknowledge at point 3 in the response letter), which would be useful to quantify. Specifically, can you combine errors due to (1) limited number of spikes per time bin and (2) limited number of time bins per trial into a single error estimate on the components of variability? This would provide a more rigorous method to decide the optimal time-bin duration.

Thank you for the clarification. The reviewer raises an important point which is a classic issue that exists for any method which uses time-bins to estimate a time varying signal (like a PSTH). Choosing small bins gives more precision in time but results in noisier or even biased estimates while having large bins improves signal to noise at the expense of time resolution. Indeed, the need for choosing the best bin size for a neuron's firing rate PSTHs has been recognized by many colleagues in the field previously with methods proposed for optimal bin selection (Shimazaki H et al 2007, Ambroggi F et al 2011). The same issue can exist when one is trying to estimate variability components in time. To address the reviewer's comment, we have added a supplementary figure (Fig S3) that illustrates the dependence of accuracy on the bin sizes and the trade-off between resolution and noise as discussed before. We have also added a cautionary note to the Result section page 9 line 4-7 while referring to the new figure.

“Obviously, the choice of the optimal time-bin also depends on how fast variability components change in time (faster changes favor smaller time-bins to maintain sufficient temporal resolution, Fig S3) similar to any other time-histogram (*34*, *35*).”
