# Supplementary material for: Stimulus presentation can enhance spiking irregularity across subcortical and cortical regions
Source: PLoS Comput Biol. 2022 Jul 5;18(7):e1010256. doi: 10.1371/journal.pcbi.1010256 (PMC9286274; doi:10.1371/journal.pcbi.1010256)
Supplement: Attachment — Submitted filename: Reviewer_comment_R3.pdf [file pcbi.1010256.s018.pdf]

Dear Editor and reviewer #2,

Thank you for taking time to provide valuable feedback and coach multiple rounds of reviewing this work. Below we addressed the final comments by the editor.

Ali Ghazizadeh

Editor's comments:

This is a minor, but I would also suggest to not use mathematical abbreviation (nψ, ϕ, nRV) in the abstract to make it more inviting. The "within-trial spike regularity" and "trial-to-trial rate variability" are much more understandable.

All abbreviations are removed from the abstract

Therefore, we suggest that you deposit the new data (vlPFC and SNr data) and code in a public depository and also provide link to the rest of data (V1, V2, MT, PMd and IpN) in the Data Availability section.

We have deposited our code and data (vlPFC and SNr) in Dryad and are providing the links to all data (previously published and new) in the Data Availability section. We have also uploaded the new data (vlPFC and SNr data) and code as a supplemental file in case it can be hosted by the journal.

Comments to the Authors:
Please note here if the review is uploaded as an attachment

We are ok with publishing the reviews.
